# Effect of seawater temperature, pH, and nutrients on the distribution and character of low abundance shallow water benthic foraminifera in the Galápagos

**DOI:** 10.1371/journal.pone.0202746

**Published:** 2018-09-12

**Authors:** Alexander F. Humphreys, Jochen Halfar, James C. Ingle, Derek Manzello, Claire E. Reymond, Hildegard Westphal, Bernhard Riegl

**Affiliations:** 1 Department of Chemical and Physical Sciences, University of Toronto Mississauga, Mississauga, ON, Canada; 2 Department of Geological Science, Stanford University, Stanford, California, United States of America; 3 Atlantic Oceanographic and Meteorological Laboratory, National Oceanic and Atmospheric Administration, Miami, Florida, United States of America; 4 Leibniz Centre for Tropical Marine Research (ZMT), Bremen, Germany; 5 Department of Geosciences, University of Bremen, Bremen, Germany; 6 Department of Marine and Environmental Sciences, Halmos College of Natural Sciences and Oceanography, Nova Southeastern University, Dania Beach, FL, United States of America; Royal Holloway University of London, UNITED KINGDOM

## Abstract

In order to help predict the effects of anthropogenic stressors on shallow water carbonate environments, it is important to focus research on regions containing natural oceanographic gradients, particularly with respect to interactions between oceanography and ecologically sensitive carbonate producers. The Galápagos Archipelago, an island chain in the eastern equatorial Pacific, spans a natural nutrient, pH, and temperature gradient due to the interaction of several major ocean currents. Further, the region is heavily impacted by the El Niño—Southern Oscillation (ENSO) and the Galápagos exhibited widespread coral bleaching and degradation following the strong ENSO events of 1982–1983 and 1997–1998. These findings are coupled with reports of unusually low abundances of time-averaged benthic foraminiferal assemblages throughout the region. Foraminifera, shelled single-celled protists, are sensitive to environmental change and rapidly respond to alterations to their surrounding environment, making them ideal indicator species for the study of reef water quality and health. Here, statistical models and analyses were used to compare modern shallow water benthic foraminiferal assemblages from 19 samples spanning the Galápagos Archipelago to predominant oceanographic parameters at each collection site. Fisher α diversity indices, Ternary diagrams, Canonical Correspondence Analysis, regression tree analysis and FORAM-Index (FI; a single metric index for evaluating water quality associated with reef development) implied a combined impact from ENSO and upwelling from Equatorial Undercurrent (EUC) waters to primarily impact foraminiferal abundances and drive assemblage patterns throughout the archipelago. For instance, repeated ENSO temperature anomalies might be responsible for low foraminiferal density, while chronically high nutrients and low aragonite saturation and low pH—induced by EUC upwelling and La Niña anomalies—likely inhibited post-ENSO recovery, and caused foraminiferal assemblages to exhibit a heterotrophic dominance in the southern archipelago. What resulted are low FI values in the southern collection sites, indicating environments not conducive to endosymbiont development and/or recovery.

## Introduction

Carbonate-producing organisms are vital to a host of shallow water marine ecosystems throughout the world, and with predictions for anthropogenic warming and its associated oceanic changes, there is a need to better understand distribution, physiology, and environmental interactions of carbonate producers ([1–3] and others). This is particularly relevant for high nutrient tropical systems, which produce sediments more suggestive of extra-tropical environments. These environments potentially represent natural laboratories for the study of ancient depositional environments, as well as future environmental conditions [4]. Among shallow water carbonate producers, benthic foraminifera are of particular relevance. These unicellular eukaryotic protists, commonly housed within external calcium carbonate or agglutinated tests (shells), are among the most common organisms found within the carbonate sediments of all major reef systems on the planet [3, 5–7]. The distribution of these ecologically sensitive marine organisms is driven by a host of interacting environmental factors including temperature, pH, salinity, nutrients, water motion, light intensity, depth, sediment texture, food, substrate, and taphonomic processes ([7–9] and others). Additionally, many species thrive within specific ecological niches [7, 10] and exhibit rapid responses to changing biotic and abiotic factors on temporal and spatial scales ([7–9] and others). As a result of these sensitive biophysical interactions, benthic foraminifera are widely considered to be primary indicators of the health of reefal environments, and useful tools for interpreting environmental change [11, 12]. This physiochemical sensitivity makes benthic foraminifera potentially highly vulnerable to temperature and nutrient anomalies associated with periodic disturbance events like the El Niño—Southern Oscillation (ENSO) [7]. This is particularly true in the Galápagos Archipelago, a moderate to high nutrient, high CO_2_, tropical environment in the eastern tropical Pacific (ETP), often strongly impacted by ENSO. Interest in the Galápagos is magnified by reports of a near absence of benthic foraminifera in its shallow water environments, relative to other ETP locations [13, 14]. Here, we use statistical models and analyses to investigate the interaction between shallow water benthic foraminifera of the Galápagos and the major local oceanographic parameters. Of particular relevance is the notion of time averaging, which is the mixing of grains of different ages prior to permanent burial in the geologic record [77]. Hence, any analyses on shallow water assemblages must be assessed not according to individual events, but rather to the long-term oceanographic conditions in which the sediments developed. In summary, this manuscript serves to highlight the environmental forces driving the diversity and distribution patterns of benthic species within Galápagos foraminiferal assemblages, and to uncover potential causes for the low foraminiferal abundances. Additionally, we discuss future changes in shallow water foraminiferal abundance under a global change scenario.

### Regional oceanography

**Fig 1:**
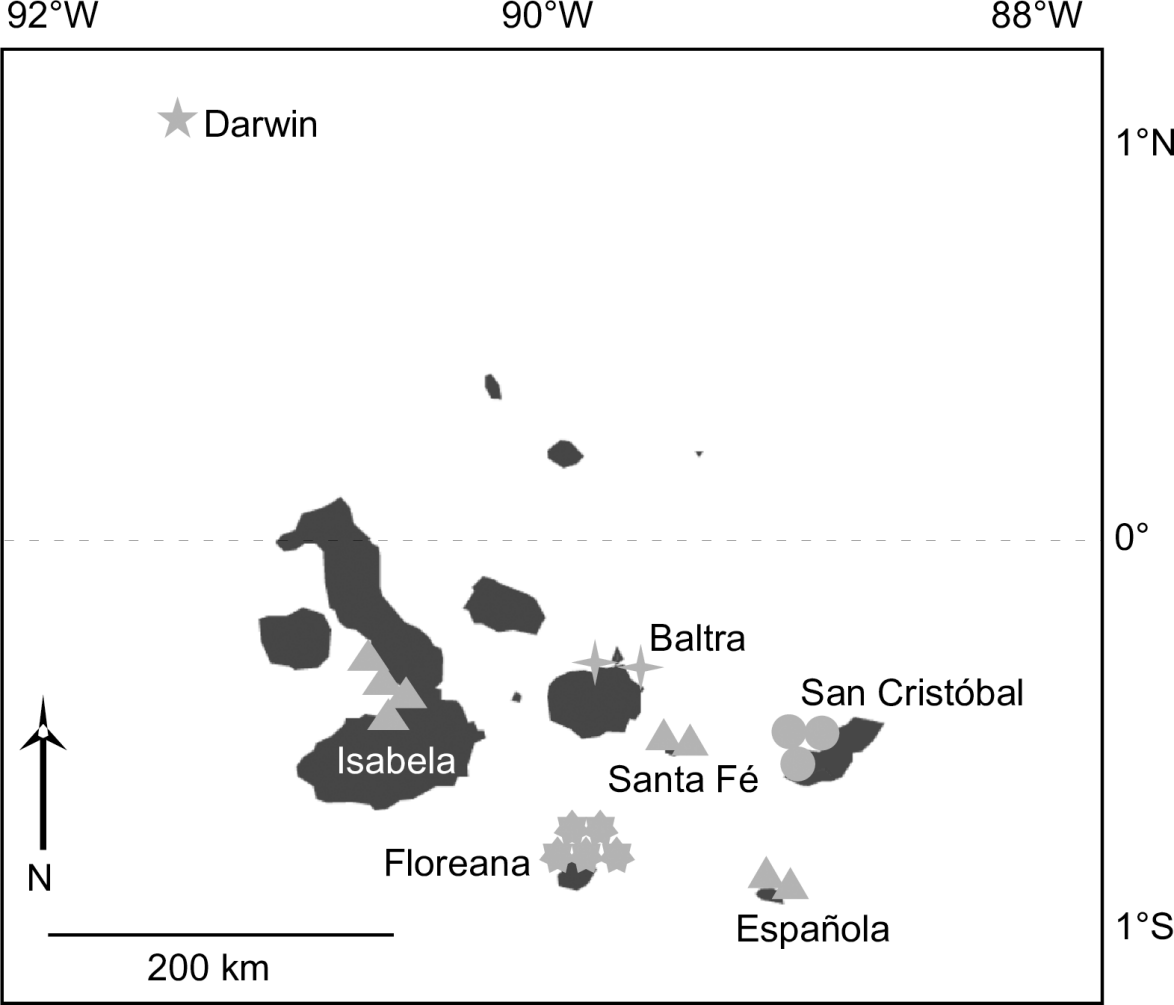
Map showing collection islands discussed in this study. Map details relative locations of samples represented by respective symbols within cluster analysis (Fig 2).

The Galápagos Archipelago (Fig 1) lies roughly 1000 km due west from the Ecuadorian mainland (Between 1°40’N-1°25’S and 89°15’W-92°00’W; [15], straddling the equator in the center of a system of currents and countercurrents [16], which cause the region to span a climatic and oceanographic transition zone with resultant variable physio-environmental conditions [17]. The southern Galápagos are directly influenced by the equatorial undercurrent (EUC) which shoals from the west, resulting in elevated nutrient levels, from mesotrophic conditions in the southeastern archipelago to eutrophic conditions at Isabela [13, 14], as well as chronically depressed pH throughout the southern islands [18]. As a direct result of this high nutrient and high CO_2_ EUC, the southern Galápagos contains the highest natural ambient CO_2_ and lowest aragonite saturation (Ω_arag_) of any modern tropical surface ocean [18]. Further, the Galápagos are strongly influenced by ENSO, a climatic and oceanic event that recurs approximately every 3–7 years. The El Niño phase of ENSO is associated with higher than normal ocean temperatures in the region, while the La Niña phase brings periods of higher-than-normal nutrient and low pH upwelling from the EUC [19]. Fluctuations in EUC and ENSO force the species and habitats in the Galápagos to face cyclical shifts in climate [19], which have had dramatic effects on the carbonate producing species of the region. For example, the stronger-than-average 1982–1983 and 1997–1998 ENSO events resulted in widespread, heat-related coral degradation throughout the southern islands [20]. Likely, foraminifera would be similarly impacted as corals [7], but no such information exists yet for the Galápagos region.

## Methodology

128 samples were collected between 1 and 46 meters water depth (see Table 1 for depth details) using SCUBA and Van Veen grabs, from seven sites spanning the geographic extent of the archipelago and stored in plastic collection bags. In the lab, samples were washed of organic material using filtered water over a 2mm and 63μm sieve stack to split the samples into gravel and sand fractions, respectively. Washed samples were dried in an oven at 50°C and the bulk sand fraction of each sample was further split (using a sediment splitter) into subsamples for foraminiferal picking (no benthic foraminifera were identified in the gravel fraction). Foraminifera were picked using ultra-fine brushes from sediment picking trays and placed in 60-cell cardboard micropaleontology slides for identification. Species identifications were performed using McCulloch [24–26], Cushman and McCulloch [27–30], and Lalicker and McCulloch [31] as primary references, as well as a number of other sources (see references [23, 32–42]). Work in the Galapagos was permitted by the authorities of Parque Nacional Galapagos (permit # 030-13PNG) and conducted under the auspices of the Charles Darwin Research Station. Furthermore, the field studies in this project did not involve endangered or protected species.

**Table 1:** In situ and satellite-derived environmental parameters used in this analysis. Sample sites showing depth, calculated Fisher α indices, species richness, FORAM-Index values, aragonite saturation (Ω_arag_), pH, and calculated mean (μ), and mean anomaly (μ_.An) for Chlorophyll-a (Chl), sea surface temperature (SST), and sea surface salinity (SSS) from satellite data for July 2002—December 2014. Mean: mean of monthly values; Mean Anomaly: per-month mean of all monthly anomalies over all months (monthly anomalies: annual mean of mean monthly values minus each monthly value). Samples: **DAR**: Darwin; **BAL**: Baltra; **ES**: Española; **SF**: Santa Fé; **IS**: Isabela; **SC**: San Cristóbal; **FL**: Floreana.

Sample	Lat	Long	Depth	Fisher α	Species	FORAM	μChl	μSST	μSSS	Ω_arag_	μChl.An	μSST.An	μSSS.An	No. of	pH
no.	(DD)	(DD)	(m)	indices	richness	Index	(mg/m^3^)	(°C)	(PSU)		(mg/m^3^)	(°C)	(PSU)	specimens	
DAR-B-43	1.673564	-91.992128	13	19.19	57	4.16	0.21	25.56	33.54	3.20	0.04	3.32	0.38	318	8.07
BAL-1	-0.488667	-90.270583	4	18.24	53	2.22	0.36	23.80	34.11	2.90	0.12	2.77	0.15	315	7.94
BAL-2	-0.488667	-90.270583	4	21.39	60	2.58	0.36	23.80	34.11	2.90	0.12	2.77	0.15	316	7.94
SF-7	-0.804000	-90.037900	4	17.93	53	2.23	0.35	23.48	34.25	3.18	0.10	2.52	0.22	308	7.97
SF-13	-0.805520	-90.034133	29	14.51	48	1.87	0.35	23.48	34.25	3.18	0.10	2.52	0.22	331	7.97
SC-33	-0.849350	-89.560917	26	10.95	37	1.57	0.49	23.44	34.24	2.67	0.17	2.73	0.22	310	7.90
SC-35	-0.850633	-89.568833	27	2.52	12	1.44	0.49	23.44	34.24	2.67	0.17	2.73	0.22	292	7.90
SC-48	-0.885283	-89.607867	30	7.94	30	1.40	0.49	23.44	34.24	2.67	0.17	2.73	0.22	339	7.90
ES-59	-1.344117	-89.649233	14	16.23	52	1.65	0.30	23.53	34.40	3.15	0.11	2.58	0.25	337	8.03
ES-63	-1.345133	-89.656050	19	15.07	48	1.99	0.30	23.53	34.40	3.15	0.11	2.58	0.25	304	8.03
FL-96	-1.217083	-90.430950	34	3.29	15	1.72	0.33	23.41	34.25	2.83	0.07	2.04	0.18	312	7.91
FL-97	-1.215400	-90.428183	28	4.75	20	1.45	0.33	23.41	34.25	2.83	0.07	2.04	0.18	316	7.91
FL-102	-1.213900	-90.425933	31	8.33	31	1.44	0.33	23.41	34.25	2.83	0.07	2.04	0.18	336	7.91
FL-105	-1.216333	-90.423850	17	1.07	6	1.92	0.33	23.41	34.25	2.83	0.07	2.04	0.18	293	7.91
FL-117	-1.228400	-90.415400	31	7.81	30	1.18	0.33	23.41	34.25	2.83	0.07	2.04	0.18	312	7.91
IS_EB-121	-0.656333	-91.197683	18	15.47	48	1.63	2.01	22.96	34.23	2.45	0.52	2.17	0.24	329	7.88
IS_EB-145	-0.590583	-91.095050	46	16.85	52	1.61	2.01	22.96	34.23	2.45	0.52	2.17	0.24	352	7.88
IS_EB-148	-0.577883	-91.103900	42	12.95	47	1.72	2.01	22.96	34.23	2.45	0.52	2.17	0.24	475	7.88
IS_UB-149	-0.401533	-91.226917	42	15.25	48	1.71	2.01	22.96	34.23	2.45	0.52	2.17	0.24	317	7.88

The preservation of foraminiferal tests tends to be higher within shallow water carbonate environments than in siliciclastic and organic-rich settings, so the sediment collection sites for this project were selected for their high carbonate sediment production—a commonly used methodology for foraminiferal analysis [21–23]. In addition to higher foraminiferal abundance, these settings are also typically characterized by superior test preservation, compared to organic-rich and siliciclastic settings [21–22]. Benthic foraminifera have been reported to be nearly absent from shallow water carbonate rocky reef settings in the Galápagos [13], so sample collection for this project focused on similar soft substrate (white sand) carbonate production sites (identified using satellite imagery) [14]. However, even with these sampling criteria, unusually low foraminiferal abundances (Avg. 0.7% of total carbonate fraction following removal of terrigenous data) obtained from sediment thin section point count analysis on the 128 samples at 300 counts-per-sample (See [14] for details on sediment thin section methodology), and poor benthic foraminiferal test preservation throughout the Galápagos, made picking and species identification challenging. Thus, in order to obtain statistically significant numbers of benthic foraminifera (300+ tests per sample), test collection concentrated on samples with sediment thin section point counts indicating test presence (88 samples; [14]). From this sample subset, species identification was completed at each sampling site for samples with a picking rate of greater than 15 tests per hour. For Darwin, Baltra and Santa Fé, which had few samples to choose from (5–7 samples), test-collection was attempted for all samples within these sites. This methodology resulted in 19 samples, representing 7 collection sites spanning the Galápagos (Fig 1).

FORAM-Index (FI) was calculated for averaged samples at each island where samples had been analyzed, as a proxy for the general state of coral reef health throughout the Galápagos Archipelago [11–12]. FI is used to determine whether water conditions within marine habitats are capable of supporting algal symbiosis, and to assess the impact of environmental stressors on coral habitats [12]. The FI is based on foraminiferal shell presence, and does not rely on coral populations, which allows for rapid and relatively simple assessments of reef environments [11, 12, 43]. It is also a useful method for comparison to other sedimentological assessments of reefal habitats, such as the coral reef turn on/turn off zone (CRTTZ; [44]). FI calculation relies on the relative abundances of symbiont-bearing, heterotrophic and opportunistic functional groups [12, 43] and is constructed using the following equation from Hallock et al. [11]:

$$FI=(10\times P_{s})+P_{o}+(2\times P_{h})$$

in which FI is the FORAM Index, P_s_ is the number of symbiont-bearing species/T, P_o_ is the proportion of the opportunistic taxa/T, P_h_ is the proportion of smaller heterotrophic taxa/T, and T is the total number of foraminifera counted [11, 12]. See Hallock et al. [11] for details.

A hierarchical agglomerative cluster analysis—Chord distance, Ward linkage—was performed on the Hellinger transformed species count data [45] from the top 28 species, representing ~75% of all benthic foraminiferal production. Hellinger transformation (*y*^*’*^_*ij*_*)* is defined by:

$${y\prime }_{ij}=\sqrt{\frac{y_{ij}}{y_{i+}}}$$

where *y* is abundance, *y*_*ij*_ is the abundance of species *j* in sample *i*, and *i+* is the sum of values over row *i* [45]. The transformation ensures that the samples are being compared according to their specific abundances, without giving undue importance to double zero counts throughout the data [45–47]. The double zero problem arises because of the nebulous interpretation of an absent species in a dataset. Species presence at two sites indicates a similarity between the sites. However, a species absence may result from the two sites lying above or below the optimal niche zone for that species or, alternatively, one site could be above and the other below the optimal niche value [46]. The decision to transform the data was made to reduce the skewing effects of the high number of zero values within our foraminiferal count results, especially due to samples with strong species dominance. Furthermore, the transformation shows the cluster analysis on a closely comparable level to the canonical correspondence analysis (CCA) results, which keep χ^2^ distances between sample sites intact, and are not affected by the double zero problem (see below).

In order to relate the distribution of the benthic foraminiferal communities to the overarching environmental constraints, a canonical correspondence analysis (CCA) was performed on the count data for the predominant 28 foraminiferal species using the ‘vegan’ package in R [48]. CCA reduces the variables to a few digestible combinations, with species, sites, and environmental variables presented in a triplot. The χ^2^ distance between sample sites is preserved, which removes the skewing effects of double-zeros in the data ([47]; See below for explanation), and species and environmental variables are represented as points and vectors on the biplots, respectively. A benefit of CCA is that the ordering of species along the canonical axes follows a pattern related to their ecological optimum [47]. Environmental parameters used in the construction of the CCA were the result of data exploration of a larger master set of regional physical and environmental data (see Appendix for master dataset), in order to remove covariant variables. These data included remotely sensed Moderate-resolution Imaging Spectroradiometer (MODIS) environmental chlorophyll-a (Chl-a as a proxy for nutrients; [49]), Seaviewing Wide Field of view Sensor (SeaWiFS) sea surface temperature (SST) data from NASA Giovanni (https://giovanni.gsfc.nasa.gov/giovanni/; [50]), sea surface salinity (SSS) estimates from The Simple Ocean Data Assimilation (SODA; [51]), as well as in situ pH and aragonite saturation data from Manzello [18], Manzello et al. [76] as well as depth (Table 1). For a description of oceanographic statistics, see Table 2. In addition to satellite oceanographic data collection, a temperature data logger was placed on Darwin Reef at a depth of 12m, from 26 November 2016 to 23 April 2017 to collect temperature data at 0.5 hour intervals in order to compare relatively long term satellite averages to short-term reefal conditions.

**Table 2:** Explanation of the major calculated oceanographic parameter.

Raw				Statistics								
Monthly Chlorophyll and Salinity Data				**Mean:** mean of monthly values								
July 2002—December 2014				**Mean Anomaly:** per-month mean of all monthly anomalies over all months)									
				(monthly anomalies: annual mean of mean monthly values minus each monthly value)								

Species richness and Fisher α diversity indices were calculated for all known foraminifera in our samples in order to better understand the relationship between foraminiferal production and diversity throughout the Galápagos. Foraminiferal tests were identified as hyaline perforate, imperforate porcellaneous, and agglutinated, according to the classification of Loeblich & Tappan (1984) [52] and plotted as ternary diagrams alongside average Fisher α indices and pH values in order to assess geographic patterns in wall structure types.

In addition to cluster analysis and CCA, a univariate regression tree analysis ([53]; ‘tree’ package in R [54]) was constructed by using samples (binned according to their respective cluster locations) as response variables, and the oceanographic controls (including sample depth) as explanatory variables. The purpose of this analysis was to look for any possible overarching environmental controls influencing the clustering of samples.

## Results

### FORAM-Index

**Fig 2:**
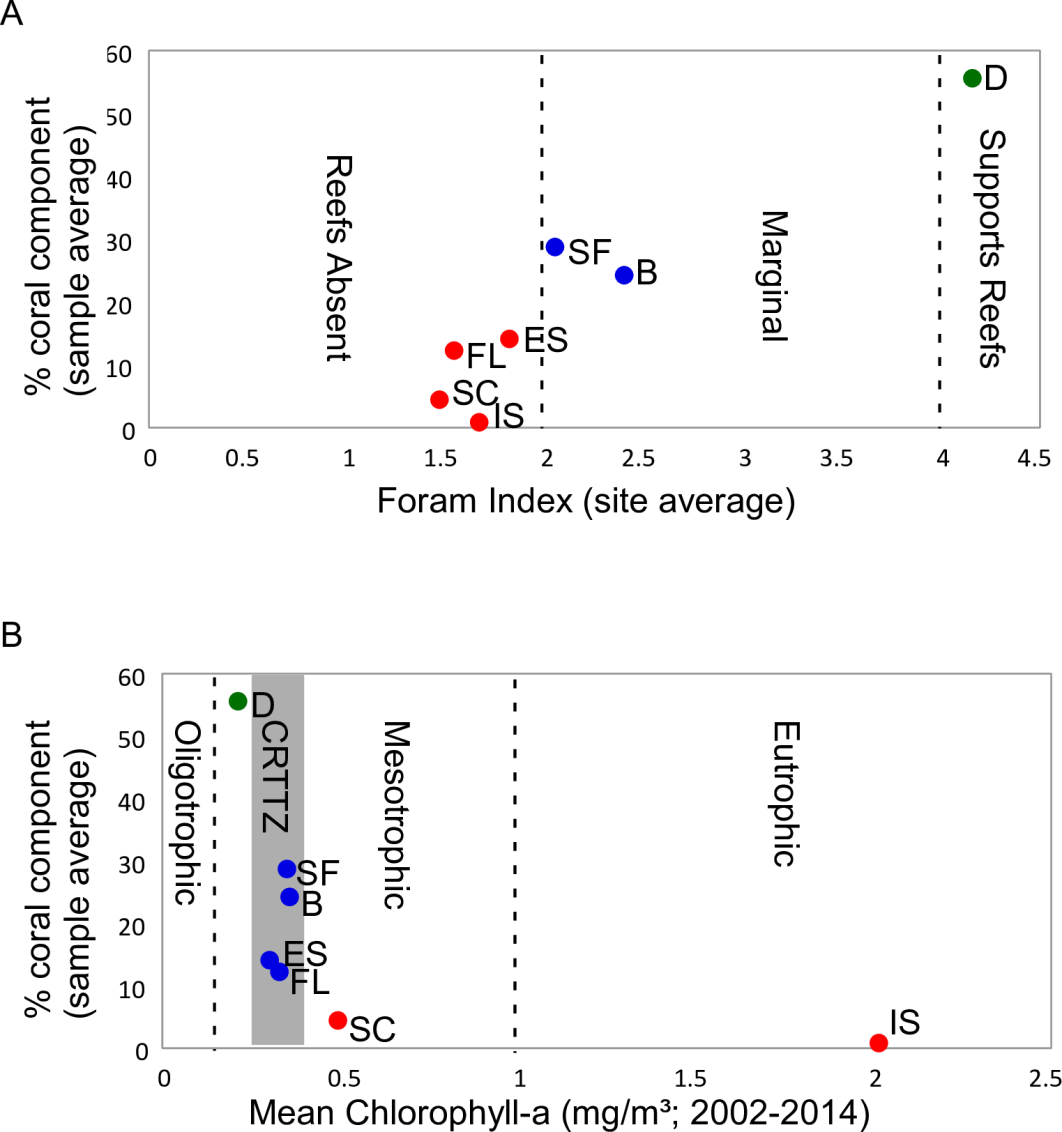
Comparisons of reef assessment analyses. (A) Average FORAM–Index values plotted against average percent coral abundance in sediments for each collection site. (B) Average mean Chlorophyll-a (nutrient proxy) plotted against average percent coral abundance in sediments for each collection site, showing relative position with respect to coral reef turn on-turn off zone (CRTTZ; [11]). Abundances based on thin section point count data for 128 samples. D, Darwin; B, Baltra; SF, Santa Fé; ES, Española; FL, Floreana; SC, San Cristóbal; IS, Isabela. Islands color coded according to strong symbiont activity and reef production (green), marginal symbiont activity and no true reef production (blue), and low symbiont production and low coral production (red) according to their respective scales.

FORAM-Index (FI) values for each site, as well as their associated ecological interpretations, are plotted in Fig 2A. Interpretations were based on the definitions of Hallock et al. [11], where FI > 4 is indicative of environmental conditions suitable for symbiotic organisms required for true reef development, as well as coral recovery. FI values between 2 and 4 indicate environments that are marginal to reef growth and not suitable for coral recovery. FI values < 2 are considered to indicate stressed environments, which are not likely to sustain reef growth. Although coral can exist in environments with FI<2, it is less common, not extensive, and not reef forming [11–12]. Fig 2A shows site-averaged FI values plotted against sample-averaged percent coral content from thin section point counts (128 samples; see Humphreys et al. [14] for details) and reveals FI values ranging from 1.2 (Floreana) to 4.2 (Darwin). The benthic foraminiferal community at Darwin falls within the low end of FI values for true reef development and post-stress recovery (green dots). FI values place Santa Fé and Baltra within the low marginal categorization, with no post-stress recovery (blue dots). All other southern sites had foraminiferal communities that indicate water conditions unsuitable for symbiotic activity (red dots; Fig 2A). Further, Fig 2A shows a photozoan sedimentary signature at Darwin (FI>4; 55% coral), a mixture of photozoan and heterozoan sediments at Baltra and Santa Fé (2<FI<4; heterozoan/photozoan transition defined as >20% corals (or others) with a majority of heterozoan carbonate producers) and diminished coral abundances in study islands with FI < 2; Española (14%), San Cristóbal (4.5%), Floreana (12%), and Isabela (0.9%; Fig 2A). Hence, FI results reveal a majority of the southern Galápagos collection sites to be unsuitable for extensive endosymbiont development (FI<2) in corals and larger foraminifera. These findings run contrary to those based on sample site mean chlorophyll values (Chl-a; [14]), which places Baltra, Santa Fé, Española, and Floreana within the coral reef turn on/ turn off zone (CRTTZ; blue dots), which demarcates the Chl-a maximum (~0.3mg/m^3^) for coral reef growth (Fig 2B). Sites with Chl-a values below the CRTTZ (green dots) readily develop reef framework, while sites with Chl-a values above the CRTTZ (red) are not conducive to coral development.

### Cluster analysis

**Fig 3:**
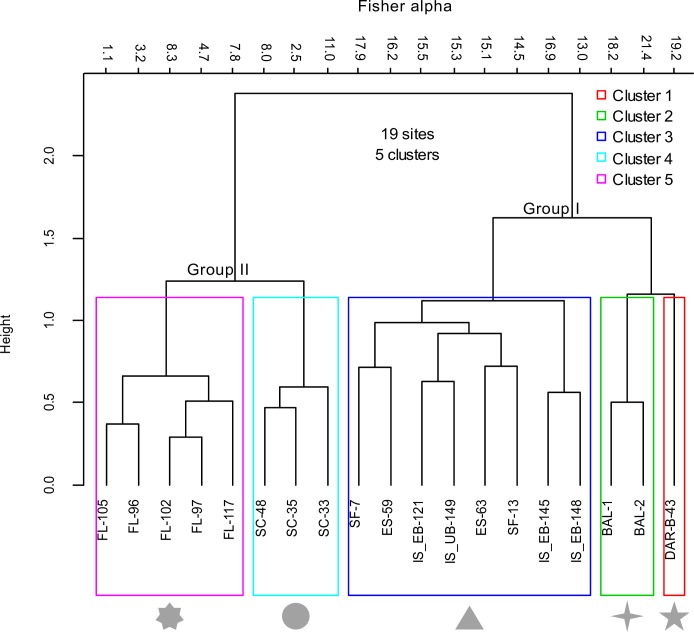
Cluster analysis. Cluster analysis (Chord Distance; Ward Linkage) on Hellinger-transformed count data for 28 foraminifera species making up top 75% of production. Sample sites from individual clusters are marked by grey symbols for corresponding plots within CCA.

Agglomerative hierarchical cluster analysis (Chord distance,Ward linkage; data Hellinger transformed), comparing the composition and abundances of the 28 predominant species throughout the Galápagos Archipelago, revealed 5 clusters, (#1–5), separated into two major groups (Group I and Group II, composed of clusters 1–3 and clusters 4–5, respectively; Fig 3).

Cluster 1 contains foraminiferal assemblages from the coral reef at Darwin Island. Cluster 2 contains samples from Baltra channel between Baltra and Santa Cruz islands. Cluster 3 contains samples from Santa Fé, Española, and Isabela. Clusters 4 and 5 are composed of samples from San Cristóbal and Floreana islands (Fig 3).

### Canonical correspondence analysis (CCA)

**Fig 4:**
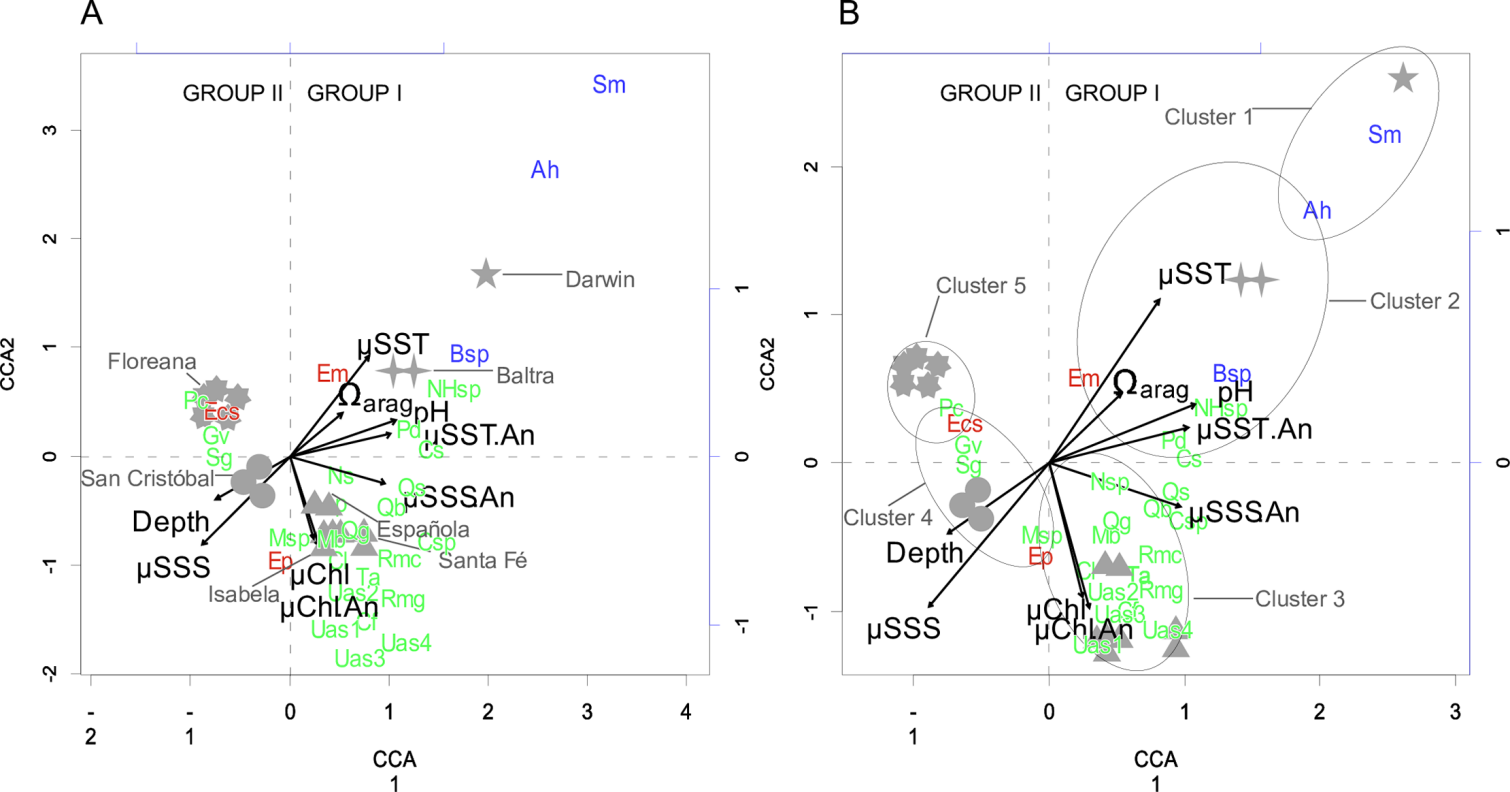
Canonical correspondence analysis (CCA) triplot results. CCA constructed from percent abundance data of 28 most abundant species, making up roughly 75% of total population of foraminifera. (A) optimal display for cluster sample (grey symbols) interpretation; (B) optimal display of foraminifera species interpretation. CCA results represent constrained ordination of foraminiferal population numbers, and not principal components analysis (PCA) of environmental variables at each site. Thus, triplots display how foraminiferal community is organized with respect to environmental parameters [47]. Environmental parameters are plotted as vectors (black), samples—labeled with their respective cluster symbols—are represented as points, and abbreviated species names (Table 1 caption) are plotted as points, color-coded according to their respective functional group affiliation (blue, symbiont bearing; red, opportunistic; green, heterotrophic). Permutation tests show high significance for CCA axes in question (S1 Fig).

Canonical correspondence analysis (CCA) results constructed from the percent abundance data of the 28 most abundant species, which make up roughly 75% of the total population of foraminifera (Table 1; for count data on all species encountered, see S1 Table (supplementary section)) were plotted as a triplot (Fig 4; for CCA permutation tests, see S1 Fig (supplementary section)). Samples were represented by their respective cluster analysis symbol (Fig 3), species were represented as abbreviations (see Table 3) and color coded according to functional group. The CCA and cluster analysis (Figs 4 and 3, respectively) revealed two major groupings along the CCA1 axis, with the higher diversity Group 1 (Clusters 1–3) plotting to the right, along positive CCA1 values, and the lower diversity Group 2 (Clusters 4–5) plotting to the left along negative CCA1 values (Fig 4).

**Table 3:** Percent component of the 28 species making up 75% of all foraminifera encountered. Abbreviations: **Ah**, *Amphisorus hemprichii***; Bsp**, *Borelis* sp.;**Cf**, *Cibicides fletcheri*; **Cl**, *Cibicides lobatulus*; **Csp**, *Cibicides* sp. (juvenile); **Cs**, *Cibicidoides schmitti*; **Ecs**, *Elphidium crispum subcrispum*; **Em**, *Elphidium macellum*; **Ep**, *Elphidium postulosum*; **Gv**, *Gypsina vesicularis***; Mb**, *Miniacina barringtonensis*; **Msp**, *Miniacina* sp.; **NHsp**, *Neohauerina* or *Hauerina* sp. (all specimens degraded); **Nsp**, *Nouria* sp.; **Pd**, *Parahauerina displicata*; **Pc**, *Poroeponides cribrorepandus*; **Qb**, *Quinqueloculina blackbeachensis*; **Qg**, *Quinqueloculina galapagosensis*; **Qs**, *Quinqueloculina suborbicularis*; **Rmc**, *Rotorbinella mira clarionensis*; **Rmg**, *Rotorbinella mira galapagosensis*; **Sm**, *Sorites marginalis*; **Sg**, *Sphaerogypsina globulus*; **Ta**, *Triloculina ashbrooki*; **Usp1**, Unidentifiable agglutinated specimens 1; **Usp2**, Unidentifiable agglutinated specimens 2; **Usp3**, Unidentifiable agglutinated specimens 3; **Usp4**, Unidentifiable agglutinated specimens 4. Samples: **DAR**: Darwin; **BAL**: Baltra; **ES**: Española; **SF**: Santa Fé; **IS**: Isabela; **SC**: San Cristóbal; **FL**: Floreana.

Species (abb.)	DAR-B-43	BAL-1	BAL-2	SF-7	SF-13	SC-33	SC-35	SC-48	ES-59	ES-63	FL-96	FL-97	FL-102	FL-105	FL-117	IS_EB-121	IS_EB-145	IS_EB-148	IS_UB-149
**Ah**	18.24	13.02	13.61	0.00	0.00	0.00	0.00	0.00	0.00	0.66	0.00	0.00	0.00	0.00	0.00	0.00	0.00	0.00	0.00
**Bsp**	4.40	1.59	0.95	0.00	0.60	0.00	0.00	0.59	0.00	3.29	0.00	0.00	0.00	0.00	0.32	1.85	0.28	0.00	0.00
**Cf**	0.63	0.00	0.63	4.22	7.85	1.93	0.34	0.29	5.04	9.54	0.00	0.00	0.00	0.00	0.64	4.32	4.26	5.67	4.91
**Cl**	0.00	0.00	0.00	0.00	3.02	2.25	0.34	0.59	2.97	1.97	0.00	0.32	0.89	0.00	0.00	0.93	0.28	0.42	0.92
**Csp**	3.77	0.32	1.27	3.57	3.02	0.00	0.00	0.00	2.08	4.28	0.00	0.00	0.00	0.00	0.00	2.78	1.14	2.10	0.92
**Cs**	4.72	0.00	0.32	1.95	1.51	0.00	0.00	0.29	1.78	0.33	0.00	0.32	0.00	0.00	0.96	1.85	0.85	1.47	1.23
**Ecs**	0.00	0.00	0.00	1.62	3.32	0.32	0.00	0.29	0.59	2.96	7.37	14.56	11.61	4.10	24.36	0.00	5.11	2.31	1.23
**Em**	0.00	30.16	6.01	0.00	0.60	0.00	0.00	9.73	0.89	0.66	5.77	6.33	3.27	0.00	13.78	1.85	2.84	3.78	2.45
**Ep**	0.00	0.32	1.27	0.32	3.02	33.44	33.90	24.78	11.87	3.95	0.64	1.58	4.46	3.41	3.85	33.02	12.78	0.63	20.86
**Gv**	0.00	0.00	0.63	0.65	0.00	5.14	5.48	1.77	0.00	0.00	0.00	5.06	6.25	0.00	2.24	0.31	0.00	0.63	0.00
**Mb**	0.00	0.63	2.22	0.32	0.30	0.64	3.08	1.18	11.28	0.00	0.00	0.32	1.19	0.00	0.00	4.01	0.00	0.21	0.00
**Msp**	0.00	0.00	0.32	0.32	5.14	0.00	8.22	6.49	1.48	1.32	0.00	0.63	2.98	0.00	0.32	3.09	0.57	0.00	0.00
**NHsp**	0.00	5.71	10.13	3.25	1.51	0.00	0.00	0.00	5.04	0.66	0.00	0.00	0.00	0.00	0.00	0.00	0.28	0.00	0.00
**Nsp**	2.83	0.00	0.00	0.65	0.60	0.32	0.00	1.18	0.00	0.99	3.21	0.00	1.79	0.00	0.00	0.62	2.27	5.25	0.31
**Pd**	0.00	3.81	6.65	1.95	2.42	0.64	0.00	0.29	1.78	0.66	0.00	0.00	0.00	0.00	1.60	0.00	0.57	0.63	0.92
**Pc**	1.57	2.86	0.63	2.92	2.72	19.61	36.64	30.09	0.00	25.00	75.00	63.92	52.68	84.30	34.62	0.31	0.57	3.99	3.68
**Qb**	0.31	2.86	6.01	4.55	0.60	2.57	0.00	0.00	5.93	0.33	0.00	0.00	0.00	0.00	0.64	1.23	3.69	1.26	1.23
**Qg**	0.00	3.49	1.58	0.00	0.00	2.89	0.34	0.00	1.48	0.99	0.64	0.00	0.30	0.00	0.32	1.54	1.42	0.84	7.98
**Qs**	0.94	2.54	1.90	1.62	2.42	0.00	0.00	0.00	1.48	1.64	0.00	0.00	0.00	0.00	0.96	1.85	0.57	0.00	0.92
**Rmc**	5.35	0.32	0.63	13.31	8.46	3.22	0.00	0.29	4.15	3.62	0.32	0.00	0.30	0.00	2.88	6.79	4.26	1.26	2.76
**Rmg**	0.00	1.27	2.53	9.74	6.34	0.64	0.00	0.29	2.08	1.64	0.00	0.00	0.30	0.00	0.32	0.62	0.28	2.94	6.75
**Sm**	14.15	1.00	0.00	0.00	1.00	0.32	0.00	0.00	0.00	0.00	0.00	0.00	0.00	0.00	0.00	0.00	0.00	0.00	0.00
**Sg**	0.31	0.00	0.00	0.65	0.00	13.18	10.27	4.72	0.00	0.99	2.88	1.58	2.68	7.51	0.96	0.00	0.28	0.00	0.00
**Ta**	0.00	0.32	0.63	1.95	2.42	0.64	0.00	0.00	2.08	6.25	0.00	0.00	0.00	0.00	0.00	0.93	0.28	0.00	2.76
**Usp1**	0.00	0.00	0.00	0.00	0.30	0.00	0.00	2.06	0.00	0.00	0.32	0.00	0.30	0.00	0.00	0.31	3.69	7.77	0.92
**Usp2**	0.00	0.50	0.00	0.00	12.99	0.64	0.00	2.00	0.00	0.00	0.00	0.00	1.00	0.00	0.64	0.00	0.00	0.00	0.00
**Usp3**	0.00	0.00	0.00	0.00	0.00	0.00	0.00	0.00	0.00	1.64	0.00	0.00	0.00	0.00	0.00	3.40	12.50	12.39	0.00
**Usp4**	0.00	0.00	0.00	13.64	0.00	0.00	0.00	0.00	5.64	0.00	0.00	0.00	0.00	0.00	0.00	0.00	0.00	0.00	0.00

Cluster 1, representing the reef setting at Darwin, was strongly associated with mean sea surface temperature (μSST) and aragonite saturation (Ω_arag_), particularly for the larger symbiont-bearing *Amphisorus hemprichii* and *Sorites marginalis*. Cluster 2, composed of samples from the south-central site of Baltra channel (Fig 1), showed a positive correlation between μSST, Ω_arag_ and the symbiont-bearing foraminifera *Amphisorus hemprichii*, *Borelis* sp., as well as the opportunistic *Elphidium macellum*. Instead, the heterotrophic *Cibicidoides schmitti*, *Parahauerina displicata* and fractured tests from *Neohauerina* or *Hauerina* species were (mostly) positively aligned with increased mean sea surface temperature anomaly (μSST.An) and pH. Cluster 3, composed of samples from Santa Fé, Española and Floreana, was heavily dominated by heterotrophic taxa, and showed a strong relationship with mean chlorophyll anomaly (μChl.An), mean chlorophyll (μChl) as well as a moderate mean sea surface salinity anomaly (μSSS.An) influence (Fig 4). This cluster was strongly associated with high quantities of unidentified agglutinated specimens (Usp) 1–4, the heterotrophic rotolids *Rotorbinella mira galapagosensis*, *Rotorbinella mira clarionensis*, *Miniacina barringtonensis*, *Cibicides fletcheri*, *Cibicides lobatulus*, as well as the miliolids *Quinqueloculina galapagosensis*, and *Quinqueloculina blackbeachensis* (Fig 4). It should be noted that Usp 1–4 were entered as a group into the statistical analyses due to the high abundance of these agglutinated tests (5% of total contribution) and their removal would have resulted in a skewing of the statistical results. Cluster 4, comprising samples from San Cristóbal, showed a strong correlation to increasing mean sea surface salinity (μSSS) and depth, a strong inverse correlation with increased μSST, Ω_arag_ and pH, and was primarily defined by the opportunistic *Elphidium postulosum* and the heterotrophic *Poroeponides cribrorepandus*, *Sphaerogypsina globulus* and a species of the genus *Miniacina*. Cluster 5, which included all samples from the north coast of Floreana (Fig 1), showed no positive association with any of the explanatory environmental variables tested, but indicated a negative correlation with μSSS.An (Fig 4). Cluster 5 was strongly grouped with the heterotrophic rotalid *Poroeponides cribrorepandus* with notable contributions from the rotalid *Gypsina vesicularis* and the opportunistic rotalid *Elphidium crispum subcrispum*.

### Foraminiferal diversity and total contribution

**Fig 5:**
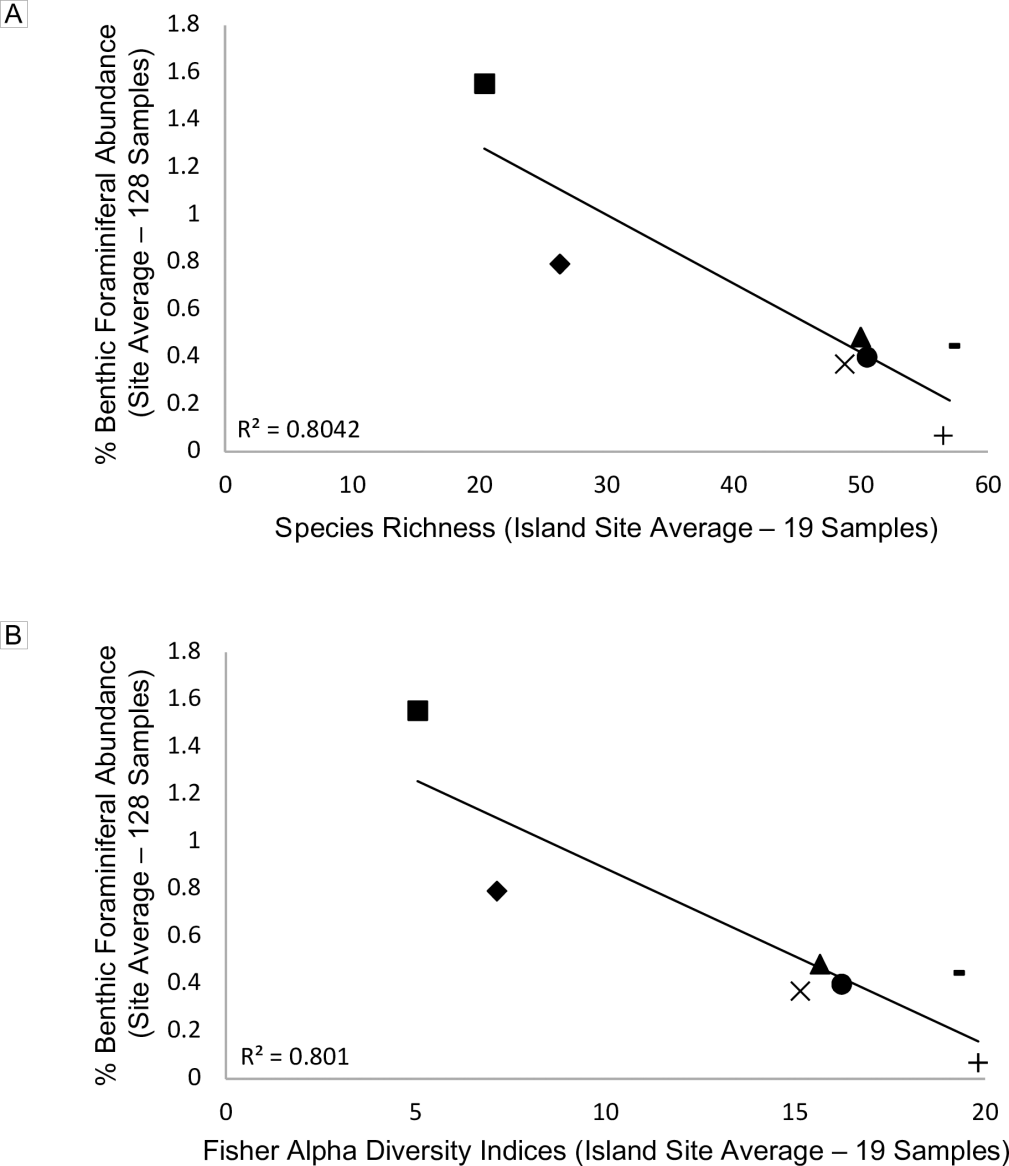
**Average percent benthic foraminifera composition plotted against species richness (A) and Fisher α diversity (B).** Plots show an inverse correlation between foraminiferal production and species richness / diversity. Islands are as follows: + Baltra;—Darwin; ● Santa Fé; ▲Española; × Isabela; ♦ San Cristóbal; ■Floreana.

Foraminiferal species richness and Fisher α diversity indices, plotted against abundance data from thin section point counts (see Humphreys et al. [14] for details), are represented in Fig 5A and 5B, respectively. Both, species richness and Fisher α indices showed strong negative correlations with respect to total foraminiferal production at each site. For instance, Baltra island foraminifera, with an average richness of 56.5 species (range: 53–60) represented an average contribution of 0.07% to total carbonate production at that site, while Floreana island foraminifera, with an average richness of 20.4 species (range: 6–31) represented an average contribution of 2.84% to total carbonate production at that site. It should be noted that Darwin Island foraminifera, which exhibited as many species as Baltra taxa, had a total foraminiferal production amounting to 0.5% of total carbonate sediment, which is comparable to the southern island sites of Santa Fé, Española, and Isabela (Fig 5). Further, diversity aligns with the clusters in the cluster analysis, with the highest sample diversity in Cluster 1 and Cluster 2 samples, moderate diversity in Cluster 3 samples, and lowest mean diversity in Group II Cluster 4 and Cluster 5 samples (Fig 3-top).

### Ternary diagrams of wall structure types

**Fig 6:**
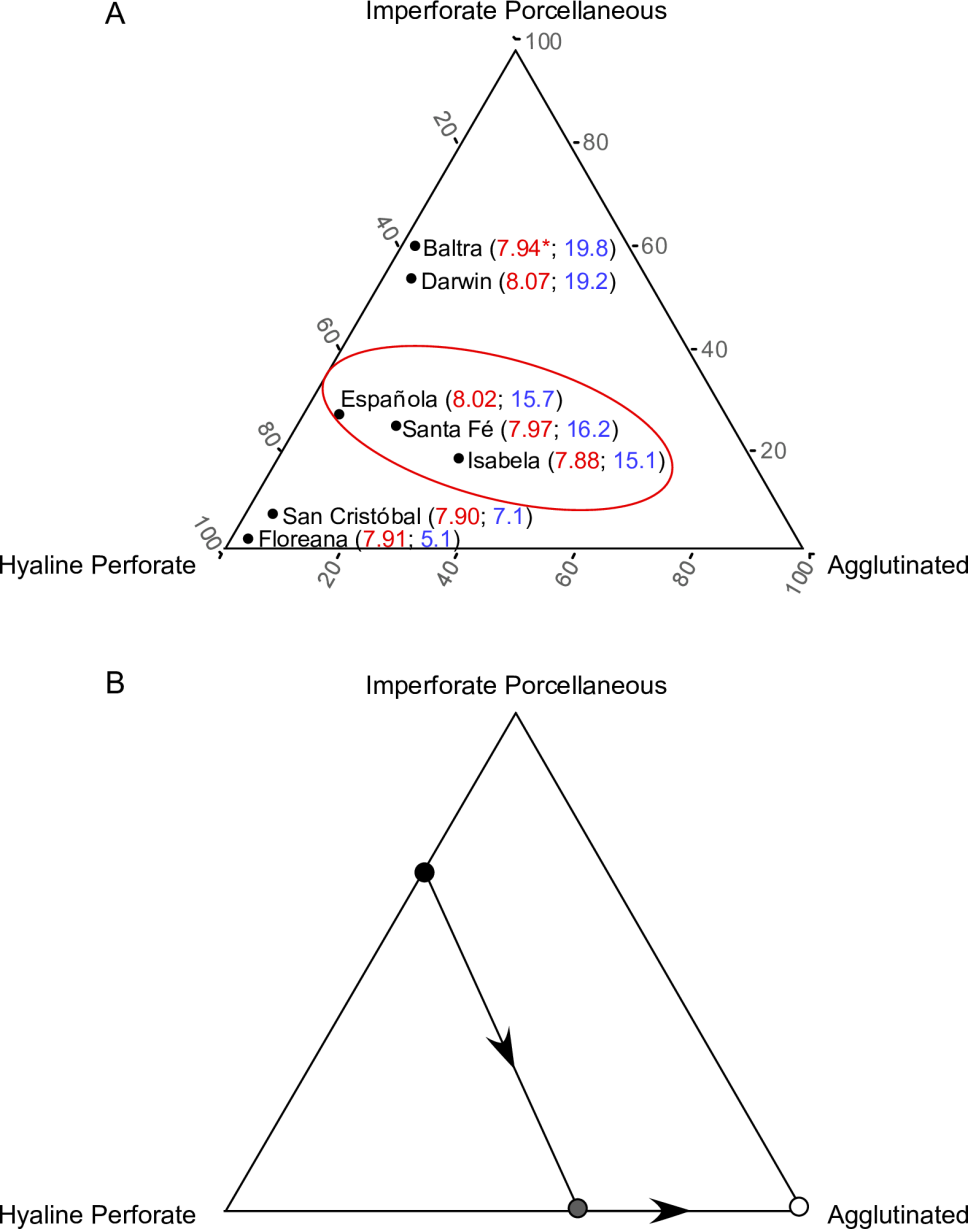
Ternary diagrams for major test structure categories of Loeblich & Tappan (1984) [52]. (A) average compositions of foraminifera from each sampled island, including mean pH (red) and mean Fisher α indices (blue), revealing a general increase in hyaline forms along declining pH and Fisher α indices gradients as well as shift toward higher agglutinated content at Española, Santa Fé and Isabela islands with decreasing pH (circled). (B) Ternary representation of a shift from calcareous to agglutinated dominance along a declining pH gradient as represented in Dias et al. [75]. *Baltra pH values are inferred from regression of all site data.

Samples from Darwin and Baltra were comosed of a majority of porcellaneous forms, with few agglutinated species. San Cristóbal and Floreana were heavily dominated by hyaline foraminifera with few agglutinated taxa (Fig 6A). Falling between these endmember groups, samples from Santa Fé, Española, and Isabela contained a majority hyaline species and follow a line of increasing agglutinated test material (Fig 6A-circled). Agglutinated test percentages were higher at the island of Santa Fé (Avg 17%; range: 15–20%) than the surrounding southeastern collection sites, and highest in two samples from Isabela (47% and 59%, respectively). In general, there was a transition toward higher rotalid dominance along declining pH and Fisher α diversity gradients (Fig 6A).

### Darwin reef temperature

**Fig 7:**
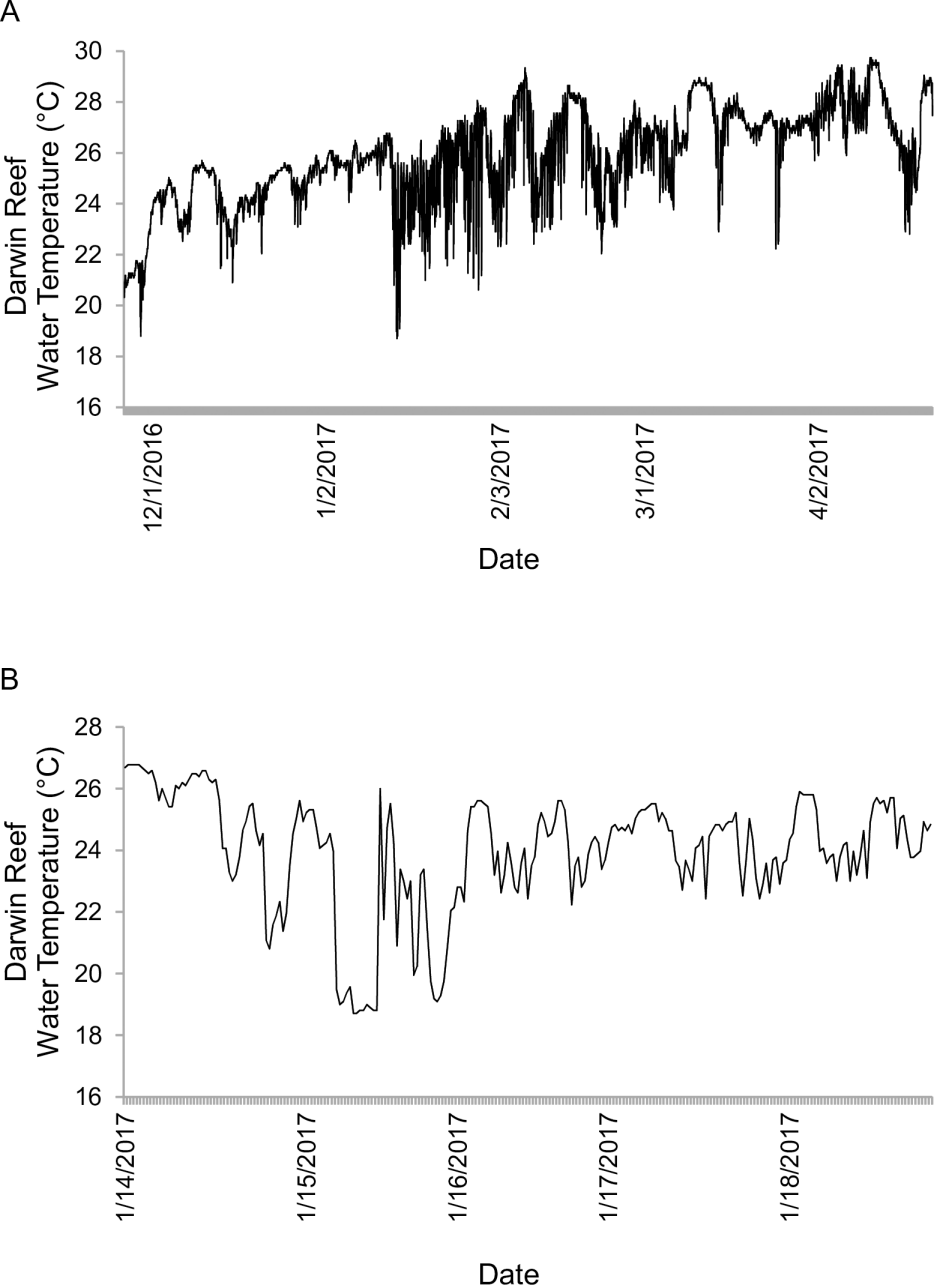
Graphs of in situ logger temperature data from Darwin reef taken at a depth of 12m. (A) Full temperature data set from 26 November 2016–18 April 2017. (B) Detail of temperature fluctuations from 14 Jan– 19 Jan 2017, showing strong temperature depression resulting from localized shoaling of deeper water masses into reef environment.

Fig 7 shows five months of in situ temperature at a depth of 12m at 0.5 hour intervals. With an average temperature 25.9°C (compared to 25.6°C for 12 year satellite average), Darwin reef showed a tropical signature, according to the SST categorizations of Betzler et al. [55]. However, Fig 7A reveals a significant temperature variability—Min 18.7°C; Max 29.7°C—over the collection period, periodically drawing the reefal setting into warm-temperate conditions. The temperature anomaly of January 14 to 19, 2017 (Fig 7B) identified the reef setting at Darwin as subjected to cooler water periods, followed by warm temperate conditions, for days at a time, which forces the tropical biofacies in the region to experience periodic temperature stresses.

### Regression tree analysis

**Fig 8:**
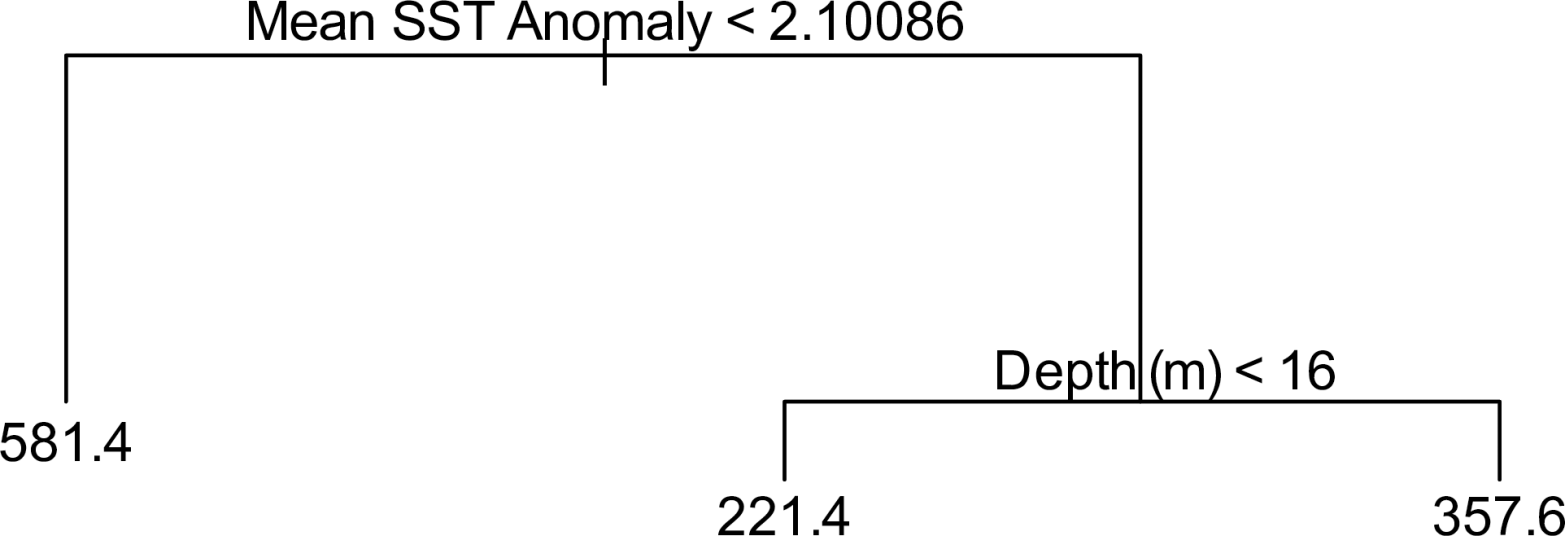
Univariate regression tree analysis. Plot shows the dominant controls on the cluster distribution in cluster analysis. The plot was produced by modeling the response variables (binned results of the cluster analysis) against the explanatory variables (oceanographic controls and sample depths. The resulting dendrogram reveals long-term average temperature anomalies (Mean.Anomaly..SST) as the dominant oceanographic influence of cluster splits, followed by sample depth.

The univariate regression tree of the major environmental parameters revealed mean sea surface temperature anomaly (μSST.An) as well as depth at each (collection) island to be the most prominent grouping variable of foraminiferal community types. In other words, changes in foraminiferal communities throughout the Galápagos samples seemed to be most strongly influenced by μSST.An, as well as collection depth (Fig 8).

## Discussion

While foraminifera in our Galápagos sediment samples were unusually rare for the Eastern Pacific, the analysis of the encountered assemblages suggested strong environmental influences that separated the northern, more tropical, parts of the archipelago, from the southern. Thus, the overall distribution of foraminifera follows general trends also observed in other calcifiers in the Galápagos ([20, 76] and others) but their rarity is clearly indicative of an at best marginal environment for these globally important carbonate producers.

### Interpretation of cluster analysis and CCA

The close overlap among cluster analysis groupings, site diversity, and CCA (species and site distributions) demonstrated a clear and unambiguous pattern of environmental influence on time-averaged Galápagos foraminiferal assemblages (Figs 3 & 4). This was particularly evident along the CCA1 axis, which divided two overriding cluster groups (Group I, Cluster1–3; Group II, Clusters 4–5; Fig 4). Long-term warmer mean water temperature (μSST), higher average aragonite saturation (Ω_arag_), pH, and average SST anomalies (μSST.AN; a proxy for Holocene El Niño variation in the region) predominantly influenced the major symbiont taxa in the low-diversity shallow waters of Darwin and Baltra (Figs 1 & 4), which resulted in the close sample association within the cluster analysis (Fig 3). Likewise, high mean nutrient water (proxied by Chl) from eastward-shoaling equatorial undercurrent (EUC) flow, as well as average nutrient anomalies (μChl.An; a proxy for Holocene La Niña in the region) positively influenced various heterotrophic species (green in Fig 4), particularly among concentrations of agglutinated taxa, at the southern sites of Isabela, Santa Fé, and Española (Figs 1 & 4). A combination of higher long-term salinity, low mean SST, and low Ω_arag_ primarily influenced predominant heterotrophic and opportunistic taxa in San Cristóbal, while temporally stable salinity values (negatively-correlated salinity anomaly) most-strongly aligned with dominant heterotrophic and opportunistic taxa at Floreana (left cluster in Figs 1 and 4). The relatively high foraminiferal abundance (mean 1.2%) exhibited in the Group II assemblages (Group I mean: 0.35%; Fig 5) further set these sites apart within the cluster analysis and CCA. The coupling of low foraminiferal diversity with high species dominance, as well as the abundant opportunistic taxa within Floreana and San Cristóbal assemblages (Figs 3 & 5; Table 3) may indicate ecological stress at these sites. Hallock et al. [11] suggested high foraminiferal opportunism to signal stressed systems. Furthermore, Floreana CCA results may indicate some as-of-yet unidentified local oceanographic influence. Floreana samples were collected in the vicinity of Corona del Diablo, an eroding volcanic cinder cone, which provides a unique shallow water environment for a multitude of invertebrate species not found elsewhere in Galápagos—including a large fungiid coral bed [56–58].

### FORAM-Index

Benthic foraminifera within the context of the FORAM-Index (Fig 2A; [11]), did not correspond with the larger nutrient-driven trends of carbonate sediment production in the Galápagos, as outlined in Fig 2B and discussed in Humphreys et al. [14]. The latter authors found coral and coralline algae to define most of the time-averaged sedimentological variation throughout the archipelago along a clear nutrient gradient delineating the coral reef turn on-turn off zone (CRTTZ; [11]). Specifically, there was a photozoan association in the low mesotrophic-upper oligotrophic (<CRTTZ, e.g. within the reef building realm) sediments of Darwin reef, a mixed photozoan-heterozoan association in all sediments from the moderate upwelling (~CRTTZ) southeastern archipelago (Floreana to San Cristóbal; Fig 2B), and a heterozoan association in the waters of western Isabela, which is directly impacted by high nutrient EUC upwelling (Fig 4B).

In contrast to the nutrient and coral sediment relationship in Fig 2B, the FI (calculated using time-averaged foraminiferal functional groups; Fig 2A) placed a majority of the low mesotrophic southeastern sites in proximity to the strongly eutrophic Isabela site (southwest) (Figs 1 & 2A). Additionally, the FI revealed all southern islands to range from low marginal (not conducive to symbiont recovery after disturbance), to stressed (not favorable to symbiont development). By exhibiting a general rise in coral-derived sediment along an increasing FI gradient (Fig 2A), the Galápagos FI results are in general agreement with the FI trends outlined in Hallock et al. [11]. However, coral production did not follow the foraminiferal-based FI as directly as it did nutrients and the CRTTZ (Fig 2). This indicates that significant oceanographic parameters in addition to nutrients may have been at play in the distribution of foraminiferal symbiont producers, resulting in the observed alignment of Isabela FI with that of a majority of southeastern islands.

The FI, which can be used as a predictor of symbiont recovery potential [11], may shed light on the impact of ENSO on the Galápagos carbonate systems. At Darwin island, which had a FI >4 (Fig 2), high rates of coral recovery were observed following the recent 1982/83 and 1997/98 ENSO events [59]. In contrast, minimal to no recovery took place in the low FI values (<4) of the southern archipelago [20, 60] which indicates marginal to stressed systems. For example, time-averaged Santa Fé carbonates contained moderate quantities of coral-derived sediment (28%; [14]) and FI values of 2.2—indicating low marginal conditions for coral reef development with a low probability of recovery (Fig 2A). This setting had previously been shown to exhibit a strong response to recent ENSO events through extensive coral degradation [61] and a subsequent shift to a rubble, rhodolith, and sand system [62]. Hence, the regionally-high coral sedimentary signature at Santa Fé (Fig 2A) may have been symptomatic of the recent stronger-than-normal ENSO-influenced ecological shift, which caused an influx of degraded coral material into the sediments, and not representative of current coral production at the site.

FI, in concert with CCA, indicated potentially contrasting outcomes for Baltra and Darwin foraminifera, and the possibility for ecological shifting among carbonate producers at the Baltra site, akin to post-1982 Santa Fé. For instance, time-averaged foraminiferal assemblages from Baltra and Darwin followed similar trends within the CCA, with μSST and Ω_arag_ positively corresponding with dominant larger symbiont bearing taxa (Fig 4). However, while rising mean sea surface temperature is forecasted to continue this century, which could benefit these larger taxa, ocean pH and aragonite saturation are predicted to further decline in the coming decades [63], which could negate these benefits. Furthermore, the strong differences in FI between Baltra and Darwin (Fig 2A), could indicate a more tenuous scenario for symbiont bearers at the low marginal FI site of Baltra, which—like Santa Fé—plots near the FI threshold (FI = 2) for environments unsuitable for symbiont activity. Unlike Darwin, the FI values at Baltra, while shaped within the context of Holocene ENSO, indicate time-averaged foraminiferal assemblages that teeter at the limit for endosymbiont development. Hence these assemblages are increasingly unlikely to recover from anomalously strong ENSO events, which are predicted to increase in frequency [64].

There is currently no consensus for the cause of the low benthic foraminiferal representation (0.7%) within the time-averaged shallow water sediments of the Galápagos [13, 14]. However, evidence suggests that ENSO may have played a major role in the low abundance of these sensitive indicator species. While studying the effects of ENSO events on benthic foraminifera within the bank reefs of northern Bahia, Brazil, Kelmo and Hallock [7] found that environmental stress brought on by the stronger-than-normal 1997–1998 ENSO led to dramatic losses in foraminiferal density in all shallow reef environments. They concluded that the 1997 El Niño resulted in declines of symbiont bearing taxa, through a combination of elevated temperature and reduced turbidity, as well as a collapse of heterotrophic taxa due to the depression of nutrient-controlled food resources. Further, Kelmo and Hallock [7] found that La Niña-associated nutrient increases resulted in a rebound in heterotrophic taxa before other forms. These findings indicate that, while the time-averaged assemblages within our samples were shaped within the context of late Holocene ENSO variability, ENSO—particularly strong ENSO events like those in 1982–1983 and 1997–1998, which devastated corals throughout the southern Galápagos islands [20, 60], might have had similar effects on benthic foraminiferal communities in the region. The long-term patterns of low abundance among time-averaged foraminiferal populations throughout the Galápagos (including at the Darwin reef site in the far northern archipelago) hint toward chronic environmental oceanographic stress as an inhibitor of post-ENSO foraminiferal rebound—keeping overall foraminifera numbers in the Galápagos low. Additionally, the dominant μChl.An signature within the Cluster 3 CCA sites (Fig 4), which are strongly indicative of La Niña nutrient anomalies in the Galápagos, suggested a close association among these southern heterotrophic (particularly agglutinated) taxa to repeated cycles of La Niña nutrient conditions—similar to those which drove the observed heterotrophic rebound in Bahia Brazil [7]. Ultimately, these findings may help explain the geographic transition toward hyaline and/or agglutinated communities in the southern Galápagos, for these foraminiferal taxa are more resistant to the higher background nutrient and lower pH conditions.

The low abundances of time-averaged foraminifera in the northern Darwin reef sediments (avg. 0.45%; 7 samples spanning the reef) were unexpected. However, while Darwin reef is not as directly impacted by EUC oceanographic effects, it experiences peripheral EUC nutrients during La Niña, as well as the highest temperature anomalies of any Galápagos island during ENSO (Table 1). These factors would likely have had significant impacts on foraminiferal densities during repeated ENSO cycles. Additionally, high resolution in situ data logger temperature measurements, taken at a depth of 12m on Darwin reef (Fig 7A), revealed this coral-dominant environment to experience strong temperature instability through time. This indicates that the tropical [14], and higher pH, Darwin island site (8.07) contains foraminifera that are repeatedly temperature stressed, which likely contributed to the record of low (time-averaged) foraminiferal sediment abundance for the site (Fig 5).

Regression tree analysis (Fig 8) offered additional support of Holocene ENSO impacts on these time-averaged foraminiferal assemblages. Although the effects of elevated nutrients and low pH in the southern Galápagos cannot be ignored, regression tree analysis (Fig 8) indicated that foraminifera may be most affected by positive temperature anomalies over time. When incorporated into the CCA and FI findings, this regression tree analysis further supported the argument that long-term and repeated exposure to El Niño (resulting in positive temperature anomalies) served as the primary suppressor of foraminifera throughout the archipelago, while high nutrient / low pH waters in the southern sites may have hindered the recovery of some species and resulted in a dominance of heterotrophic taxa over time. This caused the resultant ‘marginal’ and ‘stressed’ FI values seen in the southern Galápagos samples. Furthermore, the results supported a previous finding of an overriding high temperature and low nutrient ENSO signal over all shallow water carbonate producers throughout the Galápagos [14].

### Benthic foraminifera under naturally suppressed pH conditions

**Fig 9:**
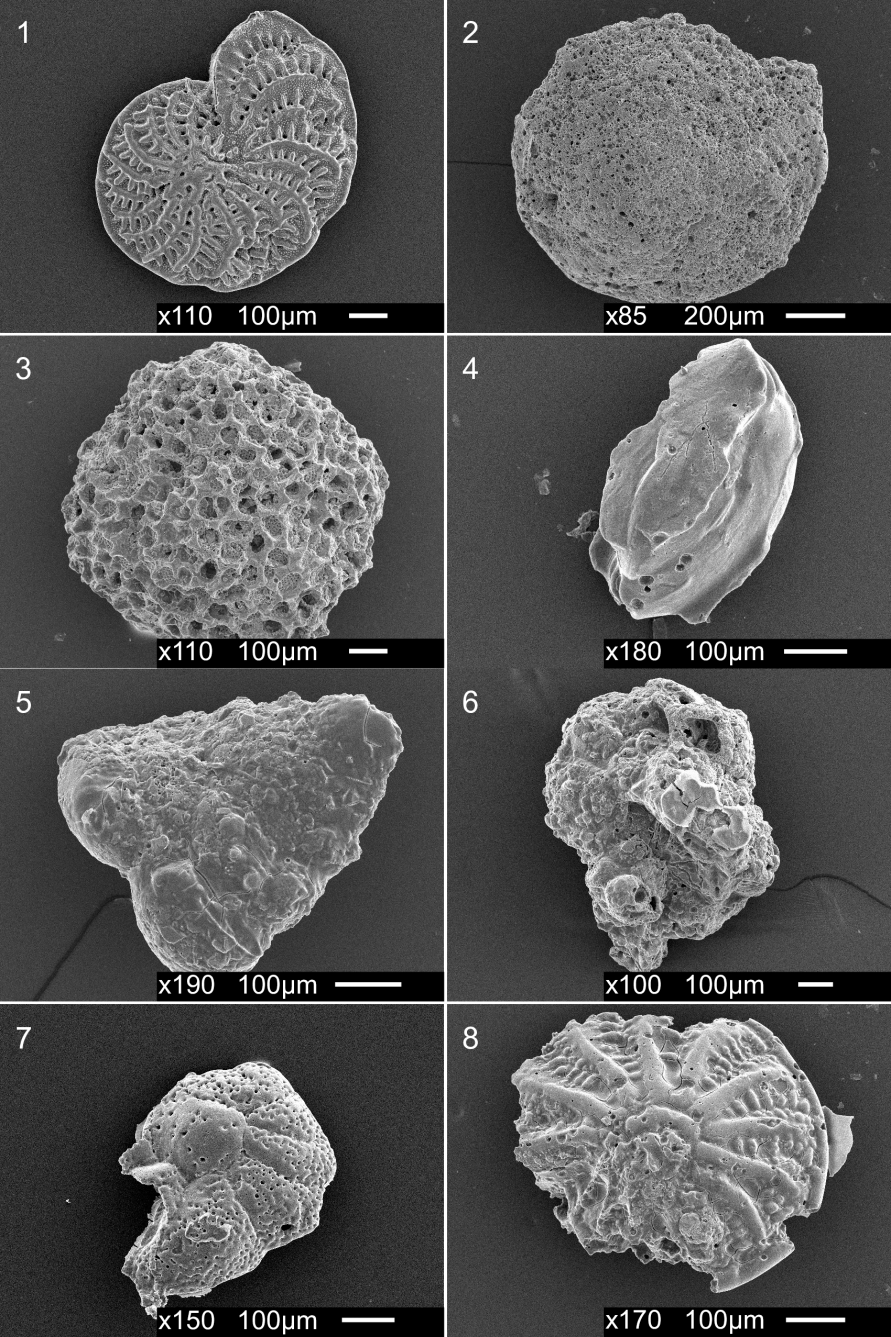
Scanning electron micrographs of select foraminifera species exhibiting varying degrees of test degradation. **1.** *Elphidium crispum subcrispum*; **2.** *Poroeponides cribrorepandus*; **3.** *Sphaerogypsina globulus*; **4.***Quinqueloculina* sp.; **5.** *Textularia* sp.; **6.** Unidentified agglutinated fragment; **7.** *Cibicides*(?) sp.; **8.** *Elphidium* sp.

The notably high proportion of broken and abraded tests in Galápagos samples was evident in all test structure types, including the large hyaline species *Poroeponides cribrorepandus* (Fig 9). However, porcellaneous and agglutinated taxa were heavily affected by degradation, with some species rendered unidentifiable due to extensive test abrasion, fracturing, and dissolution effects. For calcareous species, these dissolutions patterns likely reflected trends in test magnesium to calcium ratios (Mg/Ca), with high magnesium calcite skeletons being more susceptible to dissolution than those with low magnesium calcite skeletons [65–67]. Hence, high-Mg calcifiers would be the first to be negatively affected by a declining saturation state and ocean pH in shallow waters [66]. For calcitic foraminifera, porcellaneous benthic taxa tend to have higher Mg/Ca ratios than hyaline taxa [68]. The regionally high CO_2_/low pH extremes, from EUC and periodic La Niña anomalies (resulting in unusually intense periods of EUC upwelling), create conditions adverse to long-term porcellaneous development and may have allowed hyaline taxa or diverse agglutinated forms to proliferate. Indeed, this may explain the low densities (10% of all foraminiferal production) of the high magnesium *Quinqueloculina* (12–16 mol% [69], relative to other shallow water environments of the eastern tropical Pacific and tropical regions globally. For example, Fajemila et al. [12] reported more than 90 species of *Quinqueloculina* as well as predominances of the genera in near shore habitats of Moorea island, French Polynesia. Similarly, *Quinqueloculina* were reported to contribute more than 60% of shallow water foraminifera at La Paz, Gulf of California, Mexico [23]. The poor preservation of *Poroeponides cribrorepandus*, particularly within San Cristóbal and Floreana samples, likely stemmed from the high magnesium content of this large hyaline species. For reference, Blackmon and Todd [69] reported magnesium contents for this species at 13 mol % which were strikingly high compared to the generally well-preserved *Elphidium crispum* (3.3 mol %) found in the same Galápagos samples (low percentages of *Elphidium* specimens were found in some southern samples, however; Fig 9).

The poor preservation of agglutinated taxa in Galápagos samples was likely driven primarily by physical processes over chemical alteration of tests. In an examination of test degradation patterns in benthic foraminifera from the tropical, intertidal communities from Cleveland Bay, Australia, Berkeley and colleagues [70] found the dominant alteration process of the calcareous tests examined to be dissolution, while agglutinated tests showed a more arbitrary pathway of degradation, related to an initial loss of their organic cement coating, followed by a predominant physical-mechanical process. However, as agglutinated tests are inherently weaker than their calcareous counterparts and only a small amount of chemical degradation of the organic cements and test material is needed to undermine the entire test structure [70], it must be considered that the high CO_2_ environments of the southern Galápagos could serve to further weaken the structural integrity of agglutinated foraminifera, leading to the observed test-fracturing patterns within these samples. Ultimately, it must be stressed that carbonate dissolution is complex and potentially caused by a number of processes including corrosive sediment pore waters and bacterial destruction [71, 72] in the taphonomically active zone (TAZ), which sediments must pass through prior to permanent burial [70]. It is important for future investigations to delineate living from dead assemblages if we are to better understand the pathways to dissolution and fossilization in the region—insights which could also clarify richness and diversity patterns in Galápagos foraminifera.

The inverse correlation between foraminiferal species richness and foraminiferal abundance at each collection island (Fig 5A & 5B) was not anticipated. However, it agreed with previous studies on foraminifera in high nutrient, low pH environments [73]. For example, benthic foraminiferal assemblages along a transect of declining pH values (comparable to pH values in the Galápagos) near natural CO_2_ seeps in Papua New Guinea exhibited an observed drop in foraminiferal abundance before a decline in diversity [73].

Low pH is also known to have an additional detrimental effect on the metabolic function of some symbiont-bearing species [74]. For example, in a study of the influence of reduced pH on the growth rate of the larger symbiont bearing foraminifera *Marginopora rossi*, Reymond et al. [74] reported a drastic reduction in growth through dissolution and inhibition of precipitated calcite at the site of calcification [74]. Furthermore, rates of photosynthesis in this species decreased (primarily through a decline in endosymbiont cell density [74]) along a declining pH gradient, even at pH values similar to those observed in the southern Galápagos Archipelago. These findings may help explain the low abundance of porcellanous and larger symbiont taxa observed.

In combination with the outcome of CCA (Fig 4), the ternary diagrams reveal an additional trend among heterotrophic taxa in the assemblages from Española, Santa Fé, and Isabela islands toward a higher percentage of agglutinated species along decreasing pH regimes (Figs 4 & 6A). Similar findings were reported in a study of foraminifera within the low pH waters surrounding volcanic vents off the island of Ischia, Italy, which found a transition from calcareous forms to agglutinated taxa along a declining pH gradient ([75]; Fig 6B).

## Conclusions

This study represents the first statistical analysis of the shallow water benthic foraminiferal communities of the Galápagos Archipelago, Eastern Tropical Pacific (ETP), and their relationship to major regional oceanographic controls. Results indicate long term and repeated ENSO temperature anomalies to influence low foraminiferal density in Galápagos carbonate sediments. Naturally low levels of pH—induced by La Niña and equatorial undercurrent (EUC) upwelling—may have primarily inhibited post ENSO recovery and, in concert with EUC upwelling nutrients, resulted in heterotrophic dominance in the southern archipelago. These oceanographic conditions result in lowered FORAM-Index values in the southern Galápagos indicating environments not conducive to endosymbiont development. This further supports the well documented ENSO-induced collapse of coral communities throughout the southern archipelago following the strong ENSO events of 1982–1983 and 1997–1998. The combined ENSO-ocean acidification effect, in concert with the predicted increase in the frequency of strong ENSO [64] and declining ocean pH [76], could result in a further increase of heterotrophic foraminiferal taxa. Additionally, forecasts have been made for the decline and ‘ecological extinction’ of benthic foraminifera globally, due to declining ocean pH, by the end of the century [76]. Hence, the extremely low abundances throughout the Galápagos may signal a system already well advanced on the path towards ecological extinction with respect to foraminifera. With benthic foraminifera considered to be important indicators of environmental change, the herein-presented results help to better understand the complex interactions driving the unique foraminiferal character of the region, and advance our knowledge of and predictions for the biogeophysical implications of a high CO_2_ world.

## Supporting information

S1 Fig Permutation tests for canonical correspondence analysis (CCA) triplot results (Fig 4). Results show high significance for both CCA plots along the displayed axes (bold).
(TIF)

S1 Table Count data for all foraminifera species encountered in each sample. (PDF)
