# Supplementary figures and images for: Effect of seawater temperature, pH, and nutrients on the distribution and character of low abundance shallow water benthic foraminifera in the Galápagos
Source: PLoS One. 2018 Sep 12;13(9):e0202746. doi: 10.1371/journal.pone.0202746 (PMC6135384; doi:10.1371/journal.pone.0202746)

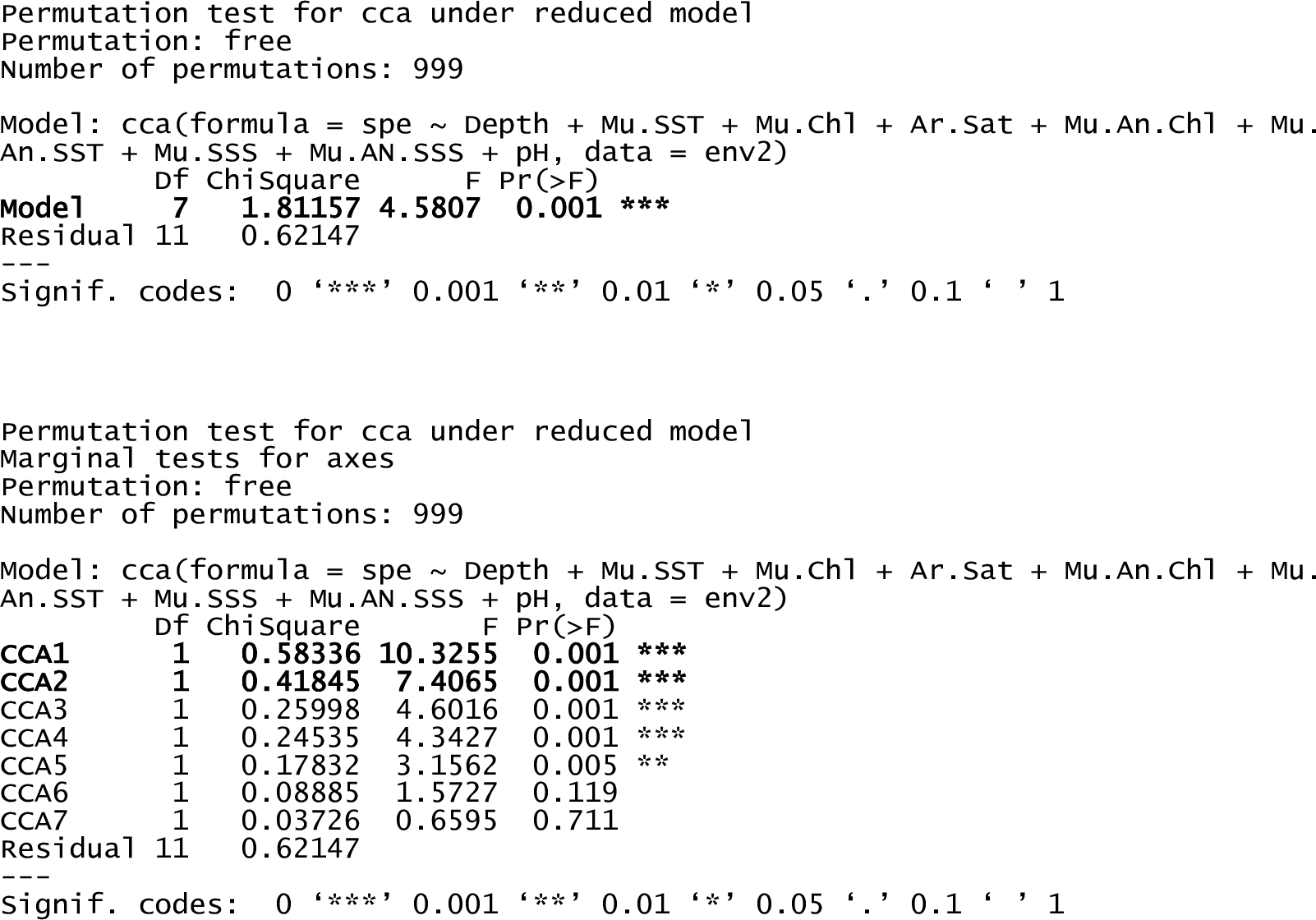

Supplement: S1 Fig — Results show high significance for both CCA plots along the displayed axes (bold). (TIF) [file pone.0202746.s001.tif]
