# Supplementary material for: Effect of seawater temperature, pH, and nutrients on the distribution and character of low abundance shallow water benthic foraminifera in the Galápagos
Source: PLoS One. 2018 Sep 12;13(9):e0202746. doi: 10.1371/journal.pone.0202746 (PMC6135384; doi:10.1371/journal.pone.0202746)
Supplement: S1 Table — (PDF) [file pone.0202746.s002.pdf]

Species Name	Author	DAR-B-43	BAL-1	BAL-2	SF-7	SF-13	SC-33	SC-35	SC-48	ES-59
Acervulina sp.	Schultze, 1854	0	0	0	0	0	0	0	0	0
Ammobaculites catenulatus	Cushman & McCulloch, 1939	0	0	0	0	0	0	0	0	0
Ammobaculites foliaceus	(Brady, 1881)	0	0	0	0	0	0	0	0	0
Amphicoryna sp.	Schlumberger in Milne-Edwards, 1881	0	0	0	0	2	0	0	0	0
Amphisorus hemprichii	Ehrenberg, 1839	58	5	0	0	0	0	0	0	0
Amphisorus sp.	Ehrenberg, 1839	0	36	43	0	0	0	0	0	0
Anomalina sp.	d'Orbigny, 1826	1	0	0	0	0	0	0	0	0
Anomalinoides porosiformis	McCulloch, 1977	0	0	2	0	0	0	0	0	1
Borelis clarionensis	McCulloch, 1977	14	7	0	0	2	1	2	1	1
Borelis sp.	Montfort, 1808	14	5	3	0	2	0	0	2	0
Buccella peruviana	McCulloch, 1977	1	0	0	0	0	0	0	0	0
Buccella sp.	Andersen, 1952	0	0	0	0	0	0	0	0	0
Buccella viejoensis	McCulloch, 1977	0	0	2	0	0	0	0	0	0
Cassidulina sp.	d'Orbigny, 1826	2	0	0	0	0	0	0	0	0
Cibicides dispars	(d'Orbigny, 1839)	0	0	0	2	0	0	0	0	3
Cibicides albidus	McCulloch, 1977	2	0	0	0	0	0	0	0	0
Cibicides fletcheri	Galloway & Wissler, 1927	2	0	2	13	26	6	1	1	17
Cibicides floridanus	(Cushman, 1918)	1	0	0	1	1	0	0	0	0
Cibicides gallowayi	Cushman & Valentine, 1930	0	0	0	0	0	0	0	0	0
Cibicides guadalupensis	McCulloch, 1977	0	0	0	0	0	0	0	0	1
Cibicides lobatulus	(Walker & Jacob, 1798)	0	0	0	0	10	7	1	2	10
Cibicides sp.	Montfort, 1808	16	1	5	11	15	0	0	0	7
Cibicides sp.	Montfort, 1808	3	0	0	0	1	0	0	0	23
Cibicidoides clarionensis	McCulloch, 1977	0	0	0	4	0	0	0	0	4
Cibicidoides schmitti	(Cushman & Wickenden, 1929)	14	0	0	6	3	0	0	1	5
Cibicidoides sp.	Brotzen, 1936	1	0	1	0	2	0	0	0	1
Cribrobaggina soccoroensis	McCulloch	1	0	0	0	0	0	0	0	0
Cribrogoësella pacifica	Cushman Mcculloch, 1939	0	1	2	0	0	0	0	5	0
Crumia albida	McCulloch, 1977	3	1	2	7	4	0	0	0	4
Crumia albionensis	McCulloch, 1977	2	0	0	15	8	0	0	0	4
Cymbaloporetta sp.	Cushman, 1928	0	0	0	0	0	0	0	0	0
Dyocibicides biserialis	Cushman & Valentine, 1930	2	0	0	1	0	0	0	0	1
Earltheeia playablancaensis	McCulloch, 1977	0	1	0	0	0	0	0	0	0
Elphidium crispum subcrispum	Cushman & McCulloch, 1940	0	0	0	5	11	1	0	1	2
Elphidium macellum	(Fichtel & Moll, 1798)	0	95	19	0	2	0	0	33	3
Elphidium postulosum	Cushman & McCulloch, 1940	0	1	4	1	10	102	99	84	40
Elphidium sp.	Montfort, 1808	0	0	0	0	0	2	0	0	0
Gaudryina pauperata	Earland, 1934	0	0	0	0	0	0	0	0	0
Glabratella melpomenensis	McCulloch, 1977	0	0	0	0	0	0	0	0	2
Glabratella semourensis	McCulloch, 1977	0	1	0	3	0	0	0	0	0
Glabrorosalina distincta	McCulloch, 1977	0	0	0	0	0	0	0	0	1
Goesella sp.	Cushman, 1933	3	3	0	0	15	1	0	1	0
Gypsina galapagosensis	McCulloch, 1977	0	0	0	1	0	1	0	5	0
Gypsina guadalupensis	McCulloch, 1977	0	0	0	0	0	0	0	0	3
Gypsina sp.	Carter, 1877	1	10	4	12	8	0	0	2	0
Gypsina vesicularis	(Parker & Jones, 1860)	0	0	2	1	0	15	16	1	0
Hauerina planiformis	McCulloch, 1977	0	1	0	0	0	0	0	0	0
Heterostegina curva	Möbius, 1880	5	0	0	0	0	0	0	0	0
Hoeglundina guadalupensis	McCulloch, 1977	1	0	0	0	0	0	0	0	0
Homotrema sp.	Hickson, 1911	0	0	0	0	0	0	0	0	0
Lagena chasteri	Millett, 1901	1	0	0	0	0	0	0	0	0
Lagenosolenia bilagenoides	McCulloch, 1977	0	0	0	0	1	0	0	0	0
Lenticulina orbicularis	(d'Orbigny, 1826)	0	0	0	0	0	0	0	0	0
Loxostomum limbatum costulatum	(Cushman, 1922)	4	6	10	2	0	0	0	0	1
Massilina robustior	Cushman & Valentine, 1930	0	2	2	0	0	0	0	0	1
Massilina socorroensis	McCulloch, 1977	0	0	0	0	0	0	0	0	0
Massilina(?) inaffecta	McCulloch, 1977	0	0	0	1	0	0	0	0	0
Melonis braithwaitensis	McCulloch, 1977	5	0	1	0	0	0	0	0	0
Miliolinella Charlesensis	McCulloch, 1977	4	0	0	0	1	0	0	0	0
Miliolinella laplataensis	McCulloch, 1977	7	0	1	0	0	0	0	0	0
Miliolinella oblonga	(Montagu, 1803)	1	0	0	0	0	0	0	0	0
Miliolinella sp. (juvenile)	Wiesner, 1931	0	0	3	1	0	0	0	0	0
Miniacina barringtonensis	McCulloch, 1977	0	2	7	1	1	2	9	4	38
Miniacina sp.	Galloway, 1933	0	0	1	1	17	0	24	22	5
Neoanomalina chincaensis	McCulloch, 1977	0	0	0	0	0	0	0	0	0
Neoconorbina clarionensis	McCulloch, 1977	1	0	0	0	0	0	0	0	0
Neoconorbina cumulata	McCulloch, 1977	0	0	0	0	0	0	0	0	0
Neoconorbina irregulariformis	McCulloch, 1977	0	0	0	0	0	0	0	1	1
Neoconorbina lapazensis	McCulloch, 1977	1	0	1	0	0	0	0	0	0
Neoconorbina sp. (juvenile)	Hofker, 1951	0	0	0	0	0	0	0	0	0
Neohauerina elongata	McCulloch, 1977	0	0	0	0	0	0	0	0	0
Neohauerina or *Hauerina* sp.	McCulloch, 1977; d'Orbigny, 1839	0	18	35	10	5	0	0	0	17
Neohauerina socorroensis	McCulloch, 1977	0	3	0	4	7	2	0	0	7
Neohauerina sp. (juvenile)	McCulloch, 1977	0	0	2	0	3	0	0	0	0
Nonionella decora	Cushman & McCulloch, 1940	0	1	1	0	3	0	0	0	1
Nouria sp.	Heron-Allen & Earland, 1914	9	0	0	2	2	1	0	4	0
Parahauerina sp.?	McCulloch, 1977	0	1	0	0	0	0	0	0	0
Parahauerina displicata	McCulloch, 1977	0	11	21	6	8	2	0	1	6
Pararosalina densiformis	McCulloch, 1977	1	0	0	0	0	0	0	0	0
Pararosalina socorroensis	McCulloch, 1977	1	0	0	0	0	0	0	0	0
Placopsilina bradyi	Cushman & McCulloch, 1939	0	1	2	1	4	0	0	0	0
Placopsilina sp.	d'Orbigny, 1850	0	2	0	0	0	1	0	0	1
Planorbulina acervalis	(Brady, 1884)	0	0	0	0	1	1	0	0	0
Planorbulina sp.	d'Orbigny, 1826	0	0	0	0	0	1	0	0	0
Planorbulinella larvata	(Parker & Jones, 1865)	1	0	0	0	0	0	0	0	0
Planorbulinella sp.	Cushman, 1927	0	1	0	0	0	0	0	0	0
Planorbulinoides retinaculata	(Parker & Jones, 1862)	0	0	1	0	0	0	0	0	0
Poroeponides cribrorepandus	Asano & Uchio, 1951	5	9	2	9	9	61	107	102	0
Psammosphaera galapagosensis	McCulloch, 1977	0	0	0	0	0	1	0	0	0
Psammosphaera parva	Flint, 1899	0	1	0	0	0	0	0	0	0

Species Name	Author	DAR-B-43	BAL-1	BAL-2	SF-7	SF-13	SC-33	SC-35	SC-48	ES-59
Pyrgo cf. *fiorei*	McCulloch, 1977	1	0	0	0	0	0	0	0	0
Quinqueloculina academybayensis	McCulloch, 1977	0	0	0	0	0	1	0	0	3
Quinqueloculina blackbeachensis	McCulloch, 1977	1	9	19	14	2	8	0	0	20
Quinqueloculina cf. *laevigata*	d'Orbigny, 1839	0	0	0	0	0	0	0	0	0
Quinqueloculina cf. *riveroae*	Bermúdez & Seiglie, 1963	1	0	0	0	0	0	0	0	0
Quinqueloculina cf. *sangrabrieliana*	McCulloch, 1977	0	1	0	0	0	0	0	0	0
Quinqueloculina contortiformis	McCulloch, 1977	0	0	0	0	0	0	0	0	0
Quinqueloculina cupicaensis	McCulloch, 1977	0	0	0	1	0	0	0	0	0
Quinqueloculina curvata	McCulloch, 1977	0	2	3	0	0	1	0	0	0
Quinqueloculina decipiens	McCulloch, 1977	0	0	0	0	0	0	0	0	0
Quinqueloculina dispar	McCulloch, 1977	0	0	0	0	0	0	0	0	0
Quinqueloculina duncanensis	McCulloch, 1977	0	2	0	0	0	0	0	0	0
Quinqueloculina galapagosensis	McCulloch, 1977	0	11	5	0	0	9	1	0	5
Quinqueloculina hancocki	McCulloch, 1977	0	0	0	0	0	1	0	0	0
Quinqueloculina heterocostata	McCulloch, 1977	0	0	0	0	0	0	0	0	0
Quinqueloculina inculcata	McCulloch, 1977	0	2	1	0	0	0	0	0	0
Quinqueloculina laguardaensis	McCulloch, 1977	0	1	0	0	0	0	0	0	0
Quinqueloculina lalibertadensis	McCulloch, 1977	0	0	0	0	0	0	0	0	0
Quinqueloculina lamarckiana	d'Orbigny, 1839	0	8	0	0	0	2	0	1	0
Quinqueloculina microstriata	McCulloch, 1977	0	0	0	0	0	1	0	0	0
Quinqueloculina microstriata gorgonaensis	McCulloch, 1977	0	0	0	0	0	1	0	8	0
Quinqueloculina neocongesta	McCulloch, 1977	0	0	1	0	0	0	0	0	0
Quinqueloculina neoreticulosa	McCulloch, 1977	0	0	4	2	0	0	0	0	0
Quinqueloculina neoreticulosiformis	McCulloch, 1977	1	2	0	0	0	0	0	0	2
Quinqueloculina opulenta	McCulloch, 1977	0	0	0	1	0	0	0	0	0
Quinqueloculina pinasbayensis	McCulloch, 1977	3	0	6	4	0	0	0	0	0
Quinqueloculina procera	McCulloch, 1977	0	0	0	2	0	0	0	0	6
Quinqueloculina prolixa	McCulloch, 1977	0	0	0	1	0	0	0	0	0
Quinqueloculina reticulosiformis	McCulloch, 1977	0	0	0	0	0	0	0	0	0
Quinqueloculina riveroae	Bermúdez & Seiglie, 1963	2	0	0	3	0	0	0	0	0
Quinqueloculina secasensis	McCulloch, 1977	0	2	0	0	0	0	0	0	0
Quinqueloculina semiquadrata	McCulloch, 1977	0	0	7	4	0	1	0	0	2
Quinqueloculina sp.	d'Orbigny, 1826	2	12	11	5	9	8	0	10	11
Quinqueloculina sp. *(juvenile)*	d'Orbigny, 1826	2	0	7	6	3	0	0	0	3
Quinqueloculina suborbicularis	d'Orbigny, 1826	3	8	6	5	8	0	0	0	5
Quinqueloculina subparkeri	McCulloch, 1977	8	0	0	5	0	0	0	0	0
Quinqueloculina taguscovensis	McCulloch, 1977	0	0	0	0	1	2	0	0	1
Quinqueloculina vulgaris	d'Orbigny, 1826	0	0	0	2	0	0	0	0	0
Quinquiloculina sangabrieliana	McCulloch, 1977	0	0	0	0	0	0	0	0	0
Reophax scorpiurus	Montfort, 1808	0	0	0	0	0	0	0	2	0
Reussella aequa	Cushman & McCulloch, 1948	0	0	1	0	1	0	0	2	0
Reussella pacifica	Cushman & McCulloch, 1948	0	0	0	0	0	0	0	0	6
Rosalina columbiensis	(Cushman, 1925)	0	0	0	0	0	0	0	0	0
Rosalina globularis	d'Orbigny, 1826	3	0	0	0	0	0	0	0	0
Rosalina micens	(Cushman, 1933)	0	0	0	0	0	0	0	0	0
Rosalina peruviana	d'Orbigny, 1839	0	0	1	0	1	0	0	0	3
Rosalina sp.	d'Orbigny, 1826	2	0	2	0	3	0	0	0	7
Rosalina vivida?	McCulloch, 1977	0	3	0	0	0	0	0	0	0
Rotorbinella mira clarionensis	McCulloch, 1977	17	1	2	41	28	10	0	1	14
Rotorbinella mira galapagosensis	McCulloch, 1977	0	4	8	30	21	2	0	1	7
Rotorbinella rosea	d'Orbigny, 1832	0	0	0	0	1	0	0	0	0
Rotorbinella turbinata	(Cushman & Valentine, 1930)	0	0	0	2	4	0	0	0	1
Rudigaudryina inepta	Cushman & McCulloch, 1939	0	0	0	0	0	0	0	0	0
Saccammina sp.	Sars, 1869	0	1	0	0	0	0	0	0	0
Scutuloris sp.	Loeblich & Tappan, 1953	0	1	1	0	0	0	0	0	0
Sigmoidella sp. (juvenile)	Cushman & Ozawa, 1928	0	0	1	0	0	0	0	0	0
Sigmoilina sp. (juvenile)	Schlumberger, 1887	0	0	0	0	0	0	0	0	1
Siphonina guadalupensis	McCulloch, 1977	13	0	2	0	0	0	0	0	0
Siphotextularia sp.	Finlay, 1939	0	0	0	0	0	1	0	0	0
Sorites marginalis	(Lamarck, 1816)	43	3	0	0	3	1	0	0	0
Sorites marginata	(Lamarck, 1816)	2	0	0	0	0	0	0	0	0
Sphaerogypsina globulus	(Reuss, 1848)	1	0	0	2	0	41	30	16	0
Sphaerogypsina sp.	Galloway, 1933	0	0	0	0	0	0	0	0	0
Spirillina darwinbayensis	McCulloch, 1977	0	0	0	0	0	0	0	0	1
Spirillina darwinbayensis	McCulloch, 1977	0	2	0	0	0	0	0	0	0
Spirillina seymourensis	McCulloch, 1977	1	0	1	0	0	0	0	0	0
Spirillina sp.	Ehrenberg, 1843	0	0	2	0	0	0	0	0	0
Spirolina arietina	(Batsch, 1791)	0	0	0	1	0	0	0	0	0
Spirolina sp.	Lamarck, 1804	1	2	3	1	0	0	0	0	0
Spiroloculina biconcava	McCulloch, 1977	0	0	0	0	0	0	0	1	0
Spiroloculina boonei	McCulloch, 1977	0	0	0	0	0	0	0	0	0
Spiroloculina charlesensis	McCulloch, 1977	0	0	0	0	0	0	1	0	0
Spiroloculina jamesbayensis	McCulloch, 1977	0	0	0	0	0	2	0	0	0
Spiroloculina ornatiformis	McCulloch, 1977	4	0	1	0	2	0	0	0	1
Spiroloculina sp.	d'Orbigny, 1826	0	0	0	1	0	0	0	0	0
Sporadotrema differens	McCulloch, 1977	1	0	0	1	0	0	0	1	0
Svratkina clippertonensis	McCulloch, 1977	13	0	1	0	0	0	0	0	0
Textularia calva	Lalicker, 1940	0	0	0	0	0	0	0	0	0
Textularia conica	d'Orbigny, 1839	0	0	0	0	0	0	0	0	0
Textularia fistula	Cushman, 1911	0	0	0	0	0	0	0	0	0
Textularia foliacea	Heron-Allen & Earland, 1915	0	0	0	0	0	0	0	0	0
Textularia schencki	Cushman & Valentine, 1930	0	0	0	0	0	0	0	0	0
Textularia scupula	Lalicker & McCulloch, 1940	0	0	0	0	0	0	0	0	0
Textularia secasensis	Lalicker & McCulloch, 1940	0	0	0	0	0	0	0	0	0
Textularia spp.	Defrance, 1824	0	0	0	0	0	0	0	0	0
Trifarina galapagoensis	McCulloch, 1977	0	0	0	0	0	0	0	0	0
Triloculina ashbrooki	McCulloch, 1977	0	1	2	6	8	2	0	0	7
Triloculina bertheliniana	(Brady, 1884)	0	1	0	0	6	0	0	0	0
Triloculina cf. *neocarinata*	McCulloch, 1977	0	0	0	0	0	0	0	0	0
Triloculina georgesensis?	McCulloch, 1977	0	0	0	0	0	0	0	0	0
Triloculina prolixa	McCulloch, 1977	0	0	1	0	0	0	0	0	0
Triloculina sp.	d'Orbigny, 1826	0	3	2	3	1	0	0	0	1
Triloculina tagusensis?	McCulloch, 1977	0	0	1	0	0	0	1	0	0
Triloculina transversestriata	(Brady, 1881)	0	1	1	0	0	0	0	0	0
Triloculina trigonula	(Lamarck, 1804)	0	0	30	1	0	0	0	0	0
Trochammina sp.	Parker & Jones, 1859	0	1	0	0	0	0	0	0	0
Unidentifiable agglutinated spp.		5	5	1	43	45	8	0	23	19
Valvulineria diversa	McCulloch, 1977	0	0	1	0	0	0	0	0	0
Total		318	315	316	308	331	310	292	339	337

Species Name	Author	FL-96	FL-97	FL-102	FL-105	FL-117	IS_EB-121	IS_EB-145	IS_EB-148	IS_UB-149
Acervulina sp.	Schultze, 1854	0	0	2	0	0	0	0	0	0
Ammobaculites catenulatus	Cushman & McCulloch, 1939	0	0	0	0	0	0	2	3	1
Ammobaculites foliaceus	(Brady, 1881)	0	0	0	0	0	0	0	1	0
Amphicoryna sp.	Schlumberger in Milne-Edwards, 1881	0	0	0	0	0	0	0	0	0
Amphisorus hemprichii	Ehrenberg, 1839	0	0	0	0	0	0	0	0	0
Amphisorus sp.	Ehrenberg, 1839	0	0	0	0	0	0	0	0	0
Anomalina sp.	d'Orbigny, 1826	0	0	0	0	0	0	0	0	0
Anomalinoides porosiformis	McCulloch, 1977	0	0	0	0	0	1	0	0	0
Borelis clarionensis	McCulloch, 1977	0	0	0	0	0	5	1	0	1
Borelis sp.	Montfort, 1808	0	0	0	0	1	6	1	0	0
Buccella peruviana	McCulloch, 1977	0	0	0	0	0	0	0	0	0
Buccella sp.	Andersen, 1952	0	0	0	0	0	0	0	1	0
Buccella viejoensis	McCulloch, 1977	0	0	0	0	0	0	0	0	0
Cassidulina sp.	d'Orbigny, 1826	0	0	0	0	1	0	0	0	0
Cibicides dispars	(d'Orbigny, 1839)	0	0	0	0	0	2	1	13	0
Cibicides albidus	McCulloch, 1977	0	0	0	0	0	0	0	0	0
Cibicides fletcheri	Galloway & Wissler, 1927	0	0	0	0	2	14	15	27	16
Cibicides floridanus	(Cushman, 1918)	0	0	0	0	0	3	0	0	0
Cibicides gallowayi	Cushman & Valentine, 1930	0	0	2	0	0	0	0	0	0
Cibicides guadalupensis	McCulloch, 1977	0	0	0	0	0	1	1	0	0
Cibicides lobatulus	(Walker & Jacob, 1798)	0	1	3	0	0	3	1	2	3
Cibicides sp.	Montfort, 1808	0	0	1	0	0	9	4	12	8
Cibicides sp.	Montfort, 1808	0	0	0	0	0	0	0	0	0
Cibicidoides clarionensis	McCulloch, 1977	0	0	1	0	0	0	1	2	2
Cibicidoides schmitti	(Cushman & Wickenden, 1929)	0	1	0	0	3	4	2	7	4
Cibicidoides sp.	Brotzen, 1936	0	0	0	0	0	2	1	0	0
Cribrobaggina soccoroensis	McCulloch	0	0	0	0	0	0	0	0	0
Cribrogoësella pacifica	Cushman Mcculloch, 1939	0	0	4	0	0	0	0	0	0
Crumia albida	McCulloch, 1977	0	0	0	0	0	1	1	2	0
Crumia albionensis	McCulloch, 1977	0	0	0	0	0	0	3	0	6
Cymbaloporetta sp.	Cushman, 1928	0	0	0	0	0	0	0	0	1
Dyocibicides biserialis	Cushman & Valentine, 1930	0	0	0	0	0	0	2	0	10
Earltheeia playablancaensis	McCulloch, 1977	0	0	0	0	0	0	0	0	0
Elphidium crispum subcrispum	Cushman & McCulloch, 1940	23	46	39	12	76	0	18	11	4
Elphidium macellum	(Fichtel & Moll, 1798)	18	20	11	0	43	6	10	18	8
Elphidium postulosum	Cushman & McCulloch, 1940	2	5	15	10	12	107	45	3	68
Elphidium sp.	Montfort, 1808	0	0	0	0	0	0	0	0	0
Gaudryina pauperata	Earland, 1934	0	0	1	0	0	0	0	0	0
Glabratella melpomenensis	McCulloch, 1977	0	0	0	0	0	0	0	0	0
Glabratella semourensis	McCulloch, 1977	0	0	0	0	1	0	0	0	0
Glabrorosalina distincta	McCulloch, 1977	0	0	0	0	0	0	0	0	0
Goesella sp.	Cushman, 1933	2	5	0	0	2	0	0	0	0
Gypsina galapagosensis	McCulloch, 1977	0	1	2	0	0	1	0	3	0
Gypsina guadalupensis	McCulloch, 1977	0	0	0	0	0	1	0	0	0
Gypsina sp.	Carter, 1877	0	0	0	1	0	2	1	9	2
Gypsina vesicularis	(Parker & Jones, 1860)	0	15	19	0	7	0	0	0	0
Hauerina planiformis	McCulloch, 1977	0	0	0	0	0	1	0	0	1
Heterostegina curva	Möbius, 1880	0	0	0	0	0	0	0	0	0
Hoeglundina guadalupensis	McCulloch, 1977	0	0	0	0	0	0	0	0	0
Homotrema sp.	Hickson, 1911	0	0	0	0	0	0	0	1	0
Lagena chasteri	Millett, 1901	0	0	0	0	0	0	0	0	0
Lagenosolenia bilagenoides	McCulloch, 1977	0	0	0	0	0	1	0	0	0
Lenticulina orbicularis	(d'Orbigny, 1826)	0	1	1	0	0	0	0	0	0
Loxostomum limbatum costulatum	(Cushman, 1922)	0	0	0	0	0	0	0	0	0
Massilina robustior	Cushman & Valentine, 1930	0	0	0	0	0	0	0	0	0
Massilina socorroensis	McCulloch, 1977	0	0	0	0	0	1	0	0	0
Massilina(?) inaffecta	McCulloch, 1977	0	0	0	0	0	0	0	0	0
Melonis braithwaitensis	McCulloch, 1977	0	0	0	0	0	0	0	0	0
Miliolinella Charlesensis	McCulloch, 1977	0	0	0	0	0	0	0	0	0
Miliolinella laplataensis	McCulloch, 1977	0	0	0	0	0	0	0	0	0
Miliolinella oblonga	(Montagu, 1803)	0	0	0	0	0	0	0	0	0
Miliolinella sp. (juvenile)	Wiesner, 1931	0	0	0	0	0	0	0	1	0
Miniacina barringtonensis	McCulloch, 1977	0	1	4	0	0	13	0	1	0
Miniacina sp.	Galloway, 1933	0	2	10	0	1	10	2	0	0
Neoanomalina chincaensis	McCulloch, 1977	0	0	0	0	0	1	0	0	0
Neoconorbina clarionensis	McCulloch, 1977	0	0	0	0	0	0	0	0	0
Neoconorbina cumulata	McCulloch, 1977	0	0	0	0	0	0	0	1	0
Neoconorbina irregulariformis	McCulloch, 1977	0	0	1	0	0	0	0	0	0
Neoconorbina lapazensis	McCulloch, 1977	0	0	0	0	0	0	0	0	0
Neoconorbina sp. (juvenile)	Hofker, 1951	0	0	0	0	0	0	0	0	2
Neohauerina elongata	McCulloch, 1977	0	0	0	0	0	0	0	0	0
Neohauerina or *Hauerina* sp.	McCulloch, 1977; d'Orbigny, 1839	0	0	0	0	0	0	1	0	0
Neohauerina socorroensis	McCulloch, 1977	0	0	0	0	1	1	0	0	1
Neohauerina sp. (juvenile)	McCulloch, 1977	0	0	0	0	0	0	1	0	3
Nonionella decora	Cushman & McCulloch, 1940	2	0	0	0	7	0	5	3	1
Nouria sp.	Heron-Allen & Earland, 1914	10	0	6	0	0	2	8	25	1
Parahauerina sp.?	McCulloch, 1977	0	0	0	0	0	0	0	0	0
Parahauerina displicata	McCulloch, 1977	0	0	0	0	5	0	2	3	3
Pararosalina densiformis	McCulloch, 1977	0	0	0	0	0	0	0	0	0
Pararosalina socorroensis	McCulloch, 1977	0	0	0	0	0	0	0	0	0
Placopsilina bradyi	Cushman & McCulloch, 1939	0	0	0	0	0	0	0	0	0
Placopsilina sp.	d'Orbigny, 1850	0	0	1	0	0	0	0	0	0
Planorbulina acervalis	(Brady, 1884)	0	0	0	0	0	0	0	0	0
Planorbulina sp.	d'Orbigny, 1826	0	0	3	0	2	0	0	0	0
Planorbulinella larvata	(Parker & Jones, 1865)	0	1	2	0	0	0	0	0	0
Planorbulinella sp.	Cushman, 1927	0	0	0	0	0	0	0	0	0
Planorbulinoides retinaculata	(Parker & Jones, 1862)	0	0	0	0	0	0	0	0	0
Poroeponides cribrorepandus	Asano & Uchio, 1951	234	202	177	247	108	1	2	19	12
Psammosphaera galapagosensis	McCulloch, 1977	0	0	0	0	0	0	0	0	0
Psammosphaera parva	Flint, 1899	0	0	0	0	0	0	0	0	0

Species Name	Author	FL-96	FL-97	FL-102	FL-105	FL-117	IS_EB-121	IS_EB-145	IS_EB-148	IS_UB-149
Pyrgo cf. *fiorei*	McCulloch, 1977	0	0	0	0	0	0	0	0	0
Quinqueloculina academybayensis	McCulloch, 1977	0	0	0	0	0	8	2	5	11
Quinqueloculina blackbeachensis	McCulloch, 1977	0	0	0	0	2	4	13	6	4
Quinqueloculina cf. *laevigata*	d'Orbigny, 1839	0	0	0	0	0	4	0	0	5
Quinqueloculina cf. *riveroae*	Bermúdez & Seiglie, 1963	0	0	0	0	0	0	0	0	0
Quinqueloculina cf. *sangrabrieliana*	McCulloch, 1977	0	0	0	0	0	0	0	0	0
Quinqueloculina contortiformis	McCulloch, 1977	0	0	0	0	2	0	0	0	0
Quinqueloculina cupicaensis	McCulloch, 1977	0	0	0	0	0	0	0	0	0
Quinqueloculina curvata	McCulloch, 1977	0	0	0	0	0	0	0	0	0
Quinqueloculina decipiens	McCulloch, 1977	0	0	0	0	0	0	0	2	0
Quinqueloculina dispar	McCulloch, 1977	0	0	0	0	0	1	0	0	0
Quinqueloculina duncanensis	McCulloch, 1977	0	0	0	0	0	0	0	0	0
Quinqueloculina galapagosensis	McCulloch, 1977	2	0	1	0	1	5	5	4	26
Quinqueloculina hancocki	McCulloch, 1977	0	0	0	0	0	0	0	0	3
Quinqueloculina heterocostata	McCulloch, 1977	0	0	0	0	1	0	0	0	0
Quinqueloculina inculcata	McCulloch, 1977	0	0	0	0	0	0	0	0	0
Quinqueloculina laguardaensis	McCulloch, 1977	0	0	0	0	0	0	0	0	0
Quinqueloculina lalibertadensis	McCulloch, 1977	0	0	0	0	0	0	0	0	6
Quinqueloculina lamarckiana	d'Orbigny, 1839	0	0	0	0	1	2	0	0	9
Quinqueloculina microstriata	McCulloch, 1977	0	1	0	0	0	0	0	0	0
Quinqueloculina microstriata gorgonaensis	McCulloch, 1977	0	3	1	0	6	0	0	0	0
Quinqueloculina neocongesta	McCulloch, 1977	0	0	0	0	0	0	0	4	0
Quinqueloculina neoreticulosa	McCulloch, 1977	0	0	0	0	0	0	0	0	0
Quinqueloculina neoreticulosiformis	McCulloch, 1977	0	0	0	0	0	0	0	0	0
Quinqueloculina opulenta	McCulloch, 1977	0	0	0	0	0	0	1	0	0
Quinqueloculina pinasbayensis	McCulloch, 1977	0	0	0	0	0	0	0	0	0
Quinqueloculina procera	McCulloch, 1977	0	0	0	0	0	7	1	0	6
Quinqueloculina prolixa	McCulloch, 1977	0	0	0	0	0	0	0	0	0
Quinqueloculina reticulosiformis	McCulloch, 1977	0	0	0	0	0	0	0	0	1
Quinqueloculina riveroae	Bermúdez & Seiglie, 1963	0	0	0	0	0	0	0	0	0
Quinqueloculina secasensis	McCulloch, 1977	0	0	0	0	0	0	0	0	0
Quinqueloculina semiquadrata	McCulloch, 1977	0	0	0	0	0	1	0	0	0
Quinqueloculina sp.	d'Orbigny, 1826	5	3	0	0	4	11	8	6	6
Quinqueloculina sp. *(juvenile)*	d'Orbigny, 1826	0	0	0	0	1	0	0	0	5
Quinqueloculina suborbicularis	d'Orbigny, 1826	0	0	0	0	3	6	2	0	3
Quinqueloculina subparkeri	McCulloch, 1977	0	0	0	0	0	0	0	0	1
Quinqueloculina taguscovensis	McCulloch, 1977	0	0	0	0	0	1	0	1	4
Quinqueloculina vulgaris	d'Orbigny, 1826	0	0	0	0	0	0	0	0	0
Quinquiloculina sangabrieliana	McCulloch, 1977	0	0	0	0	0	0	0	0	1
Reophax scorpiurus	Montfort, 1808	1	0	0	0	0	1	10	20	0
Reussella aequa	Cushman & McCulloch, 1948	0	1	0	0	0	0	0	0	0
Reussella pacifica	Cushman & McCulloch, 1948	0	0	0	0	2	0	3	2	0
Rosalina columbiensis	(Cushman, 1925)	0	0	0	0	0	3	0	0	0
Rosalina globularis	d'Orbigny, 1826	0	0	0	0	0	0	0	0	0
Rosalina micens	(Cushman, 1933)	0	0	0	0	0	0	0	1	0
Rosalina peruviana	d'Orbigny, 1839	0	0	0	0	0	0	3	0	5
Rosalina sp.	d'Orbigny, 1826	0	0	0	0	0	0	3	0	0
Rosalina vivida?	McCulloch, 1977	0	0	0	0	0	0	0	0	0
Rotorbinella mira clarionensis	McCulloch, 1977	1	0	1	0	9	22	15	6	0
Rotorbinella mira galapagosensis	McCulloch, 1977	0	0	1	0	1	2	1	14	22
Rotorbinella rosea	d'Orbigny, 1832	0	0	0	0	0	0	0	0	0
Rotorbinella turbinata	(Cushman & Valentine, 1930)	0	0	0	0	0	5	2	1	0
Rudigaudryina inepta	Cushman & McCulloch, 1939	0	0	0	0	0	1	0	0	0
Saccammina sp.	Sars, 1869	0	0	0	0	0	0	0	0	0
Scutuloris sp.	Loeblich & Tappan, 1953	0	0	0	0	0	0	0	0	2
Sigmoidella sp. (juvenile)	Cushman & Ozawa, 1928	0	0	0	0	0	0	0	0	0
Sigmoilina sp. (juvenile)	Schlumberger, 1887	0	0	0	0	0	0	0	0	0
Siphonina guadalupensis	McCulloch, 1977	0	0	0	0	0	0	1	0	0
Siphotextularia sp.	Finlay, 1939	0	0	0	0	0	0	0	0	0
Sorites marginalis	(Lamarck, 1816)	0	0	0	0	0	0	0	0	0
Sorites marginata	(Lamarck, 1816)	0	0	0	0	0	0	0	0	0
Sphaerogypsina globulus	(Reuss, 1848)	9	5	9	22	3	0	1	0	0
Sphaerogypsina sp.	Galloway, 1933	0	0	0	0	0	0	0	0	2
Spirillina darwinbayensis	McCulloch, 1977	0	0	0	0	0	0	3	0	1
Spirillina darwinbayensis	McCulloch, 1977	0	0	0	0	0	0	0	0	0
Spirillina seymourensis	McCulloch, 1977	0	0	0	0	0	0	2	3	2
Spirillina sp.	Ehrenberg, 1843	0	0	1	0	0	0	0	0	0
Spirolina arietina	(Batsch, 1791)	0	0	0	0	0	0	0	0	0
Spirolina sp.	Lamarck, 1804	0	0	0	0	0	4	0	0	0
Spiroloculina biconcava	McCulloch, 1977	0	0	0	0	0	0	0	0	0
Spiroloculina boonei	McCulloch, 1977	0	0	3	0	0	0	0	0	0
Spiroloculina charlesensis	McCulloch, 1977	0	0	0	1	0	0	0	0	1
Spiroloculina jamesbayensis	McCulloch, 1977	1	0	0	0	0	0	0	0	0
Spiroloculina ornatiformis	McCulloch, 1977	0	0	0	0	0	0	0	0	0
Spiroloculina sp.	d'Orbigny, 1826	1	0	0	0	0	0	0	0	0
Sporadotrema differens	McCulloch, 1977	0	0	1	0	0	0	0	0	0
Svratkina clippertonensis	McCulloch, 1977	0	0	0	0	0	0	0	0	0
Textularia calva	Lalicker, 1940	0	0	0	0	0	0	5	4	0
Textularia conica	d'Orbigny, 1839	0	0	0	0	0	0	11	16	0
Textularia fistula	Cushman, 1911	0	0	0	0	0	0	4	3	0
Textularia foliacea	Heron-Allen & Earland, 1915	0	0	0	0	0	0	0	2	0
Textularia schencki	Cushman & Valentine, 1930	0	0	0	0	0	0	12	21	0
Textularia scupula	Lalicker & McCulloch, 1940	0	0	0	0	0	0	0	1	0
Textularia secasensis	Lalicker & McCulloch, 1940	0	0	0	0	0	0	8	11	0
Textularia spp.	Defrance, 1824	0	0	0	0	0	0	3	4	0
Trifarina galapagoensis	McCulloch, 1977	0	0	1	0	0	0	0	0	0
Triloculina ashbrooki	McCulloch, 1977	0	0	0	0	0	3	1	0	9
Triloculina bertheliniana	(Brady, 1884)	0	0	0	0	0	0	0	2	0
Triloculina cf. *neocarinata*	McCulloch, 1977	0	0	0	0	0	1	0	0	0
Triloculina georgesensis?	McCulloch, 1977	0	0	0	0	0	0	0	0	0
Triloculina prolixa	McCulloch, 1977	0	0	0	0	0	0	0	0	0
Triloculina sp.	d'Orbigny, 1826	0	1	0	0	0	3	0	0	1
Triloculina tagusensis?	McCulloch, 1977	0	0	0	0	0	0	0	0	1
Triloculina transversestriata	(Brady, 1881)	0	0	0	0	0	0	0	0	0
Triloculina trigonula	(Lamarck, 1804)	0	0	0	0	0	0	0	0	0
Trochammina sp.	Parker & Jones, 1859	0	0	0	0	0	0	1	0	0
Unidentifiable agglutinated spp.		1	1	12	0	4	35	100	168	22
Valvulineria diversa	McCulloch, 1977	0	0	0	0	0	0	0	0	0
Total		312	316	336	293	312	329	352	475	317
